# Distinctive lipid signatures of bronchial epithelial cells associated with cystic fibrosis drugs, including Trikafta

**DOI:** 10.1172/jci.insight.138722

**Published:** 2020-08-20

**Authors:** Nara Liessi, Emanuela Pesce, Clarissa Braccia, Sine Mandrup Bertozzi, Alessandro Giraudo, Tiziano Bandiera, Nicoletta Pedemonte, Andrea Armirotti

**Affiliations:** 1 Analytical Chemistry Lab, Istituto Italiano di Tecnologia, Genova, Italy.; 2 L’Unità Operativa Complessa (UOC) Genetica Medica, Istituti di Ricovero e Cura a Carattere Scientifico (IRCCS) Giannina Gaslini, Genova, Italy.; 3 D3 PharmaChemistry, Istituto Italiano di Tecnologia, Genova, Italy.

**Keywords:** Cell Biology, Apoptosis, Chloride channels, Drug therapy

## Abstract

In recent years, a number of drugs have been approved for the treatment of cystic fibrosis (CF). Among them, newly released Trikafta, a combination of 3 drugs (VX-661/VX-445/VX-770), holds great promise to radically improve the quality of life for a large portion of patients with CF carrying 1 copy of F508del, the most frequent CF transmembrane conductance regulator (CFTR) mutation. Currently available disease-modifying CF drugs work by rescuing the function of the mutated CFTR anion channel. Recent research has shown that membrane lipids, and the cell lipidome in general, play a significant role in the mechanism of CFTR-defective trafficking and, on the other hand, its rescue. In this paper, by using untargeted lipidomics on CFBE41o- cells, we identified distinctive changes in the bronchial epithelial cell lipidome associated with treatment with Trikafta and other CF drugs. Particularly interesting was the reduction of levels of ceramide, a known molecular player in the induction of apoptosis, which appeared to be associated with a decrease in the susceptibility of cells to undergo apoptosis. This evidence could account for additional beneficial roles of the triple combination of drugs on CF phenotypes.

## Abstract 2

Untargeted lipidomics reveals unique signatures associated with cystic fibrosis drugs.

## Introduction

In the last 5 years, several studies have investigated the role of lipid composition and dynamics of the plasma membrane in the trafficking of cystic fibrosis (CF) transmembrane conductance regulator (CFTR), the anion transporter defective in CF. The role of cholesterol and ceramides, including short-chain ones (1), in the formation of CFTR clusters in the plasma membrane has been reported (2), as well as the stabilization of CFTR triggered by phosphatidylserines (3). An important contribution to CFTR stability at the cell membrane also comes from the action of flippases (4), enzymes known to regulate the movement of phospholipids across the cell membrane. Very recently, Bear and colleagues demonstrated that membrane cholesterol plays a significant role in CFTR activity (5). Despite this work clearly demonstrating a crucial role for lipid composition in CFTR trafficking and activity, to our knowledge there has not yet been an untargeted lipidomic profiling of CF-relevant cell models directed toward the whole lipidome and not selected lipid species. High-resolution liquid chromatography–mass spectrometry (LC-MS) represents a key resource for untargeted lipidomics, in that it allows the possibility of identifying and quantifying hundreds of individual lipid species in biofluids, cells, and tissues(6). Lipidomics has been applied to many different human tissues, such as brain (7, 8), liver (9), kidney (10), and lungs (11). Quite surprisingly, compared with other “omes” (12, 13), the lipidome of the human bronchial epithelium has been investigated less. Few papers describe analytical efforts in lipidomics directed toward bronchial epithelium (14), while most of the lipidomics work associated with CF research has been done on profiling and biomarker discovery in both plasma (15, 16) and BAL fluid (17, 18). At the cellular level, a successful CFTR rescue maneuver, beside promoting better folding and trafficking of CFTR, might be linked to significant changes in the overall lipid composition of the cell membranes, which could either favor or contrast the rescue itself. Several studies have partially addressed this point: a positive impact of pharmacological modulation of the messaging action of sphingolipids on CF pathology has been proposed in the recent works on the S1P signaling pathway (19, 20). On the contrary, a negative effect on CFTR half-life at the plasma membrane has been postulated for chronic coadministration of the potentiator ivacaftor (VX-770) and the corrector lumacaftor (VX-809) (21, 22). Works focused on F508del, the most frequent CFTR mutation, suggested that VX-770 could either destabilize VX-809–corrected F508del-CFTR, thus markedly increasing mutant protein turnover rate (21), or decrease the folding efficiency and metabolic stability of VX-809–rescued F508del-CFTR at the endoplasmic reticulum (ER) and post-ER compartments, thus causing reduced cell surface F508del-CFTR density and function (22). This evidence points toward a crucial role of lipid composition on CFTR physiology. On these premises, we conducted an extensive untargeted lipidomics investigation of immortalized F508del human bronchial epithelial (CFBE41o-) cells treated with some of the drugs (and combinations of drugs) currently available for patients with CF, including the recently introduced Trikafta. Trikafta consists of 2 correctors with different mechanism of action, VX-661 (tezacaftor) and VX-445 (elexacaftor), and VX-770 as potentiator (23). The aim of the current work was to extensively profile the lipidomic content of the F508del-CFTR human bronchial epithelium in relation to successful CFTR pharmacological rescue maneuvers. We hypothesized that treatment with these drugs, the improved trafficking and expression at the plasma membrane of mutant CFTR, might be associated with specific changes in the lipidomic profile of CFBE41o- cells, partially restoring or rebalancing the altered lipid composition.

## Results

**Figure 1:**
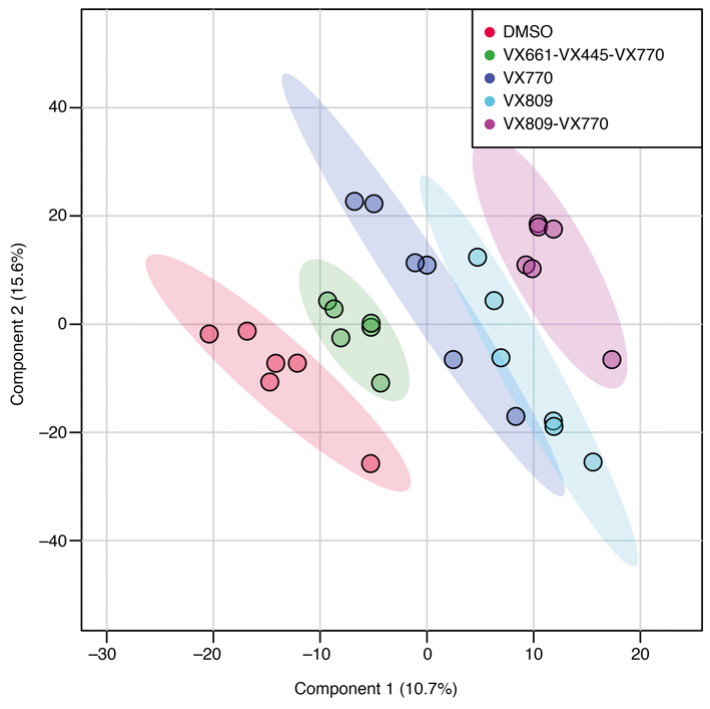
Score plot for PLS-DA analysis of CFBE41o- cells lipidome after treatment with drugs or control DMSO. Blank and QC groups are omitted for clarity.

**Figure 2:**
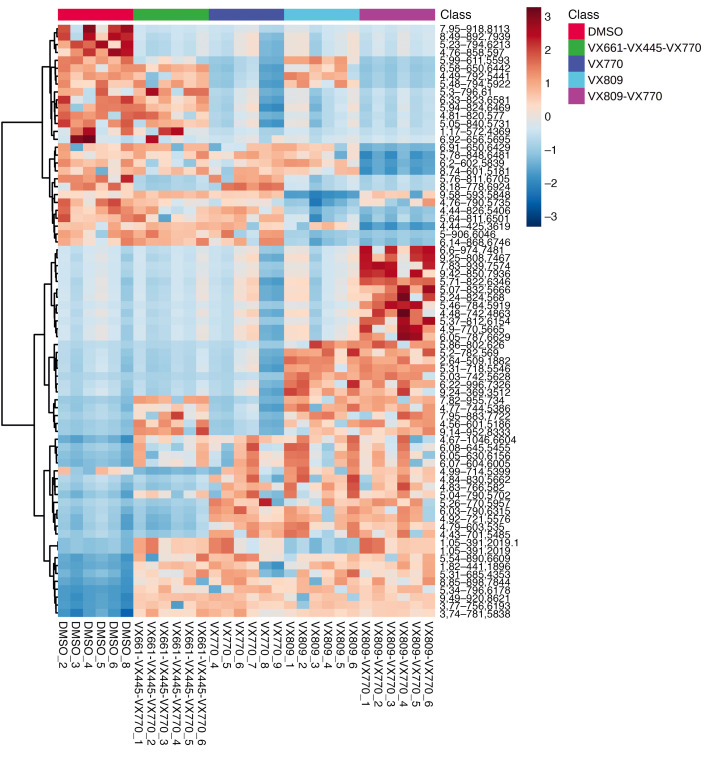
Heatmap of the clustering analysis of the CFBE41o- lipidomics sample set. Based on the top 75 PLS-DA VIP features, the samples naturally cluster into the experimental groups.

**Figure 3:**
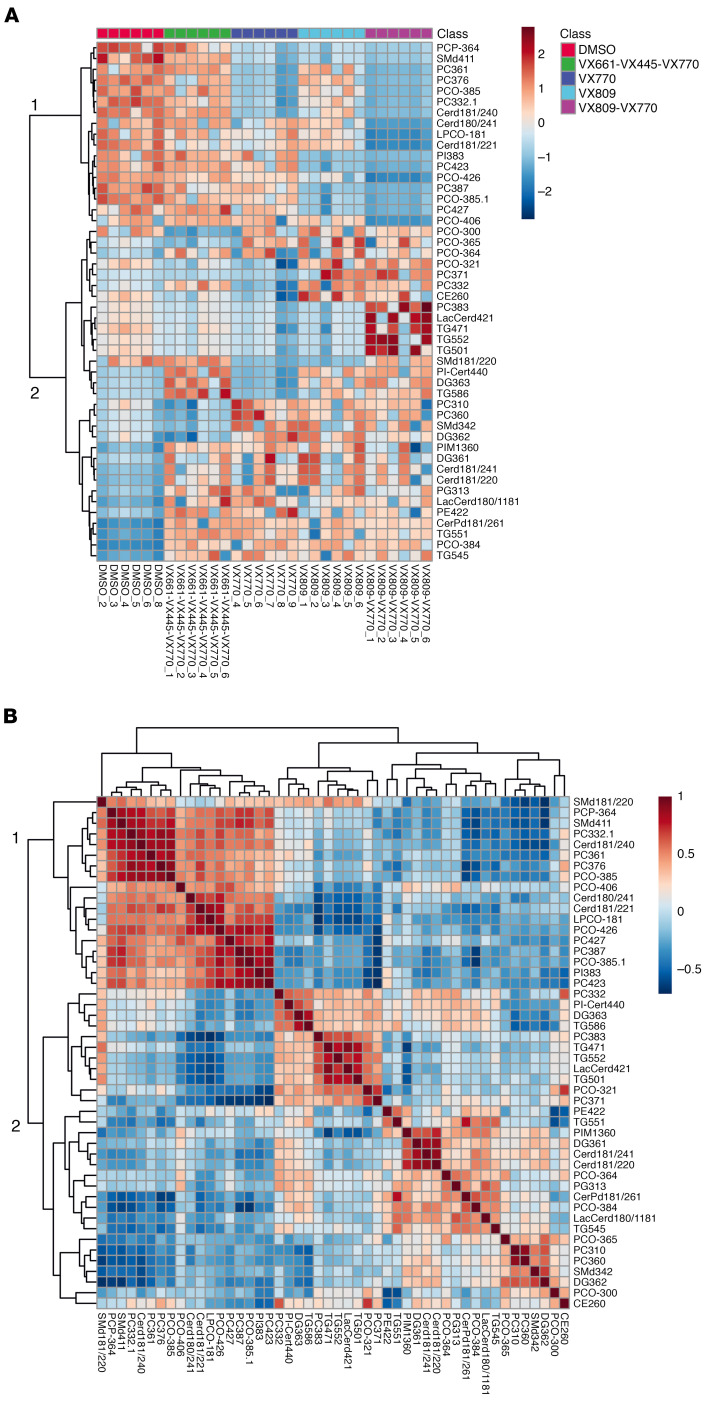
Correlation analysis for 48 lipids. (**A**) Heatmap of the clustering analysis based on the 48 PLS-DA VIP features. The samples naturally cluster into the experimental groups. (**B**) Patterns of correlation among the 48 lipids. Two main clusters of lipids were observed in the 2 data set (see 1 and 2).

Over a period of 2 months, we established and maintained 6 independent CFBE41o- cell preparations. We incubated them with the corrector VX-809 and the potentiator VX-770 as well as the drug combinations VX-809/VX-770 (Orkambi) and VX-661/VX-445/VX-770 (Trikafta). DMSO was used as control incubation. For each of these incubations, CFTR rescue was verified as described previously (24). After pelleting and lipid extraction, an extensive lipid profiling by high-resolution LC-MS was performed on these samples. Including blank, procedure blank and quality check (QC) samples, as well as dual ESI^+^/ESI^–^ data acquisition, a total of 220 analytical runs were performed. All the data generated from this sample set were then analyzed by multivariate data analysis (25), extracting all the observed features from all the experimental groups. A table with details is provided as Supplemental Data File 1 (supplemental material available online with this article; https://doi.org/10.1172/jci.insight.138722DS1). We then used both unsupervised (principal component analysis [PCA]) and supervised (partial least squares discriminant analysis [PLS-DA]) analysis methods, with the aim of identifying changes in the global lipidomic profiles. The PCA revealed a clear (but trivial) separation between blank runs and other groups (Supplemental Figure 3) but also revealed differences among the 5 experimental groups (VX-770, VX-809, VX-809/VX-770, VX-661/VX-445/VX-770, and control), particularly in PC1 versus PC3 and PC2 versus PC3 (Supplemental Figure 4). The PLS-DA revealed a clear separation between the control group lipidome (DMSO) and that of CFBE41o- cells treated with the drugs (Figure 1). We then checked our model for overfitting, as recommended for untargeted metabolomics experiments (26). Supplemental Figure 5 shows the results of this check and demonstrates that the PLS-DA model is reliable. The PLS-DA score plot, accounting for 26% of the total variability observed in the data set, shows that (a) the observed differences among the experimental groups exceed the observed variability between the different biological replicates, represented by the spreading of the dots within the same group. (b) Each drug or combination imparted peculiar changes to the cell lipidome, in such a way that each experimental group appeared separated from the others. We then extracted the data for the top 75 features contributing the most to the observed group separation (variable importance in projection [VIP] features). Supplemental Figure 6 shows the relative abundance of each feature (provisionally identified with a retention time/mass-to-charge ratio [RT_*m/z*]) in each of the experimental groups. With the same data matrix (75 features × 30 samples), we then performed a cluster analysis. The heatmap depicted in Figure 2 shows that the samples, based on these 75 features, naturally clustered into 5 separate groups corresponding to the experimental ones. We then went back to the original data with the aim to annotate these features. Each *m/z* ratio at a given RT was extracted from the LC-MS runs, checked for consistency, deconvoluted for adducts and annotated using the publicly available LIPID MAPS database (https://www.lipidmaps.org/) (27). A total of 48 of 75 features were annotated by positively assigning them to a lipid ID. Table 1 provides a list of the 48 lipids divided by lipid category, and Supplemental Data File 2 shows the final list of lipids and their peak areas in each sample. Supplemental Figure 7 shows the corresponding PCA score plot. These annotated data were then used to confirm the observed sample clusterization into the original experimental groups (Figure 3A) and to search for patterns of correlation among the annotated species (Figure 3B). Based on this analysis, 2 major clusters of lipids were observed. We thus performed a pathway analysis using the lipids belonging to these 2 clusters. As reported in Supplemental Figure 8, while lipids for cluster 1 mostly belonged to the glycerophospholipid metabolism pathway, the most enriched pathway in cluster 2 was sphingolipid metabolism. These results indicate that 2 among the most important metabolic pathways for lipids (glycerophospholipid and sphingolipid) are involved in the cellular response of F508del-CFTR–expressing CFBE41o- cells to the tested drugs. The multivariate data analysis scenario discussed thus far refers to a simultaneous comparison of all the tested groups taken all together, i.e., a general overview of the lipidomic response of the model to CFTR rescue. In order to have a picture of the distinctive changes associated with the treatment with each single drug or combination, we performed binary comparisons with each experimental group versus the control. All significant (*P* < 0.05) features were annotated (Supplemental Data File 3).

### Role of VX-770.

**Figure 4:**
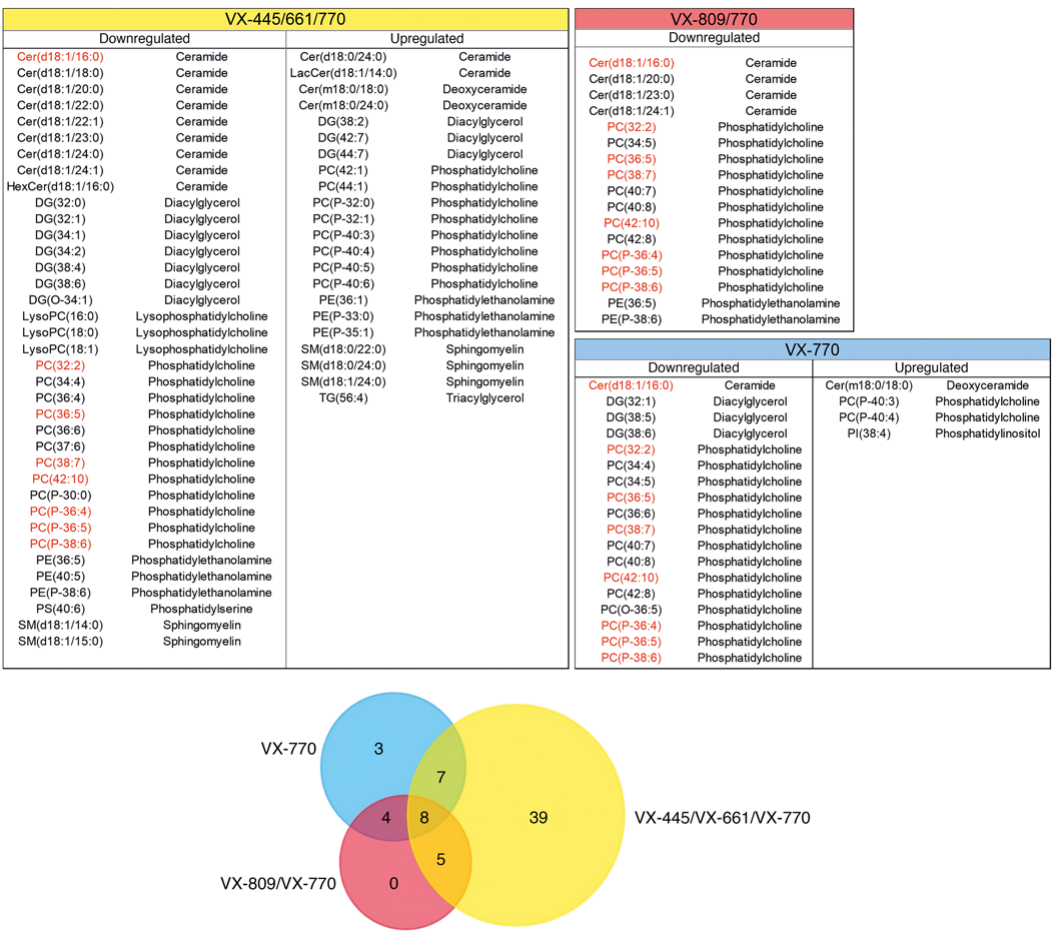
Significantly upregulated and downregulated lipids observed in the 3 VX-770 groups compared with control and corresponding Venn diagram of overlap. The 8 lipids indicated in red are at the intersection of the 3 groups. (FDR-adjusted *P* = 0.05, fold change >1.5.

Given the results reported in Figure 1, which are suggestive of an effect of VX-770 in the overall changes observed in the lipidomic signatures, we specifically investigated the molecular changes associated with this drug. Indeed, Bear and colleagues have already demonstrated (28) that VX-770 interacts with membrane lipid structures, increasing membrane fluidity. We thus interrogated our untargeted data set by comparing the annotated lipids observed in all the binary comparisons involving VX-770. Figure 4 shows upregulated and downregulated lipids and a corresponding Venn diagram, highlighting the overall data overlap between the groups. Interestingly, while treatment with Trikafta appeared to have the biggest effect on the global lipidomic changes in this model, with 39 altered molecules, 8 individual lipids (a ceramide, 4 polyunsaturated phosphatidylcholines, and 3 polyunsaturated alkenyl-phosphatidylcholines) were present at the intersection between all the 3 groups and thus appeared to be associated with VX-770. These molecules, reported in Supplemental Table 1, were downregulated in all VX-770 groups compared with control. Phosphatidylcholines are among the major components of membrane lipids (29) and have a huge effect on the mechanical and biophysical properties of the membrane itself (30, 31). Changes in their metabolism (increased turnover rate) have been associated (32) with the altered membrane recycling observed in CF. These phospholipids also constitute the largest lipid component of BAL fluid in patients with CF (33). On the other hand, PCs represent one of the largest lipid families, with 1773 individual PC species currently reported in the Lipid Maps database. It is thus difficult to exactly pinpoint the reasons for the observed downregulation of these particular 7 PCs associated with VX-770. In 2017, it was demonstrated (34) that this drug restores PC secretion mediated by ABCB4 membrane transporter. A (selective) increased secretion, perhaps mediated by other transporters, might be in line with the decrease of the individual PC we observed in the CFBE41o- lipidome. The observed downregulated ceramide also appears intriguing. Ceramides too have been associated with the properties of cell membranes (35) and with the formation and structure of lipid rafts (36). The role of these sphingolipids in CF has been explored (37): ceramides are known to accumulate in CF epithelial cells, triggering inflammation and cell death (38). The decrease of 16:0 ceramide we observed in the VX-770 groups is thus consistent with the positive effect of this drug on the CF phenotype.

### Focus on the triple combination (Trikafta).

**Figure 5:**
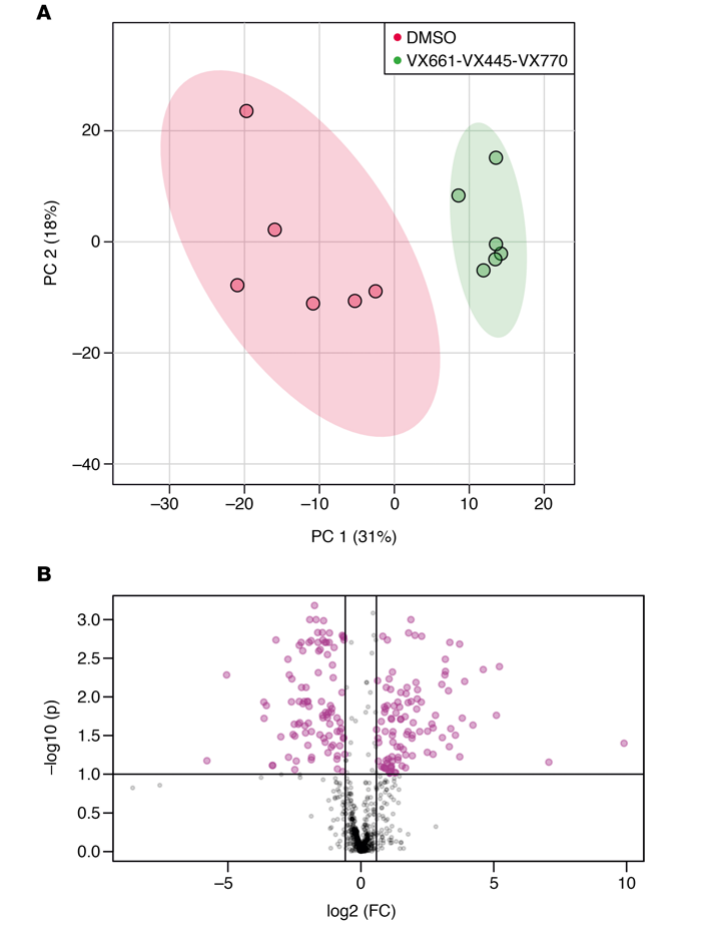
Effect of triple combination on the CFBE41o- lipidome. (**A**) PCA score plot for the control and triple combination (VX-661/VX-445/VX-770) comparison. The 2 groups are separated in unsupervised data analysis. (**B**) Volcano plot for the same comparison. The features with a fold change greater than 1.5 (FDR-adjusted *P* = 0.1) are indicated in purple along with the regulation status following treatment with Trikafta.

**Figure 6:**
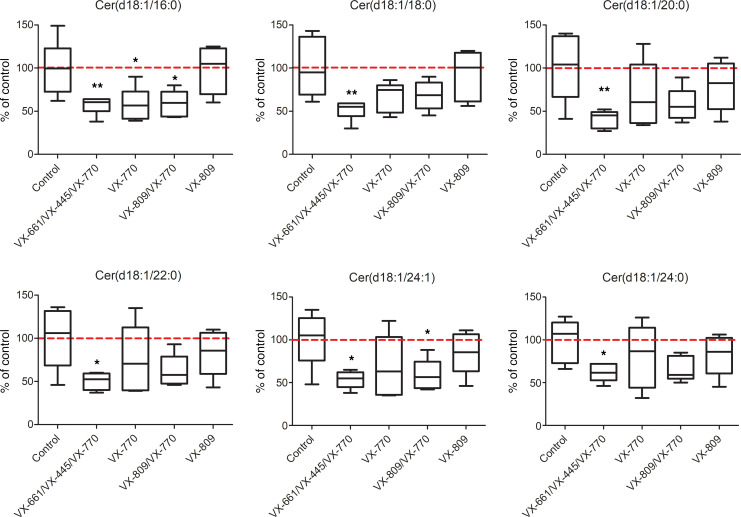
Targeted quantification of 6 ceramides in all the experimental groups. The average level of the control group is represented by the dotted red line. Box-and-whiskers plots represent the median value (inner bar), the first and third quartile (box), and the minimum and maximum value (outer bars). Data represent mean ± SEM, *n* = 5. *P < 0.05, **P < 0.01 compared with control, 1-way ANOVA test with Dunnett’s post hoc test.

**Figure 7:**
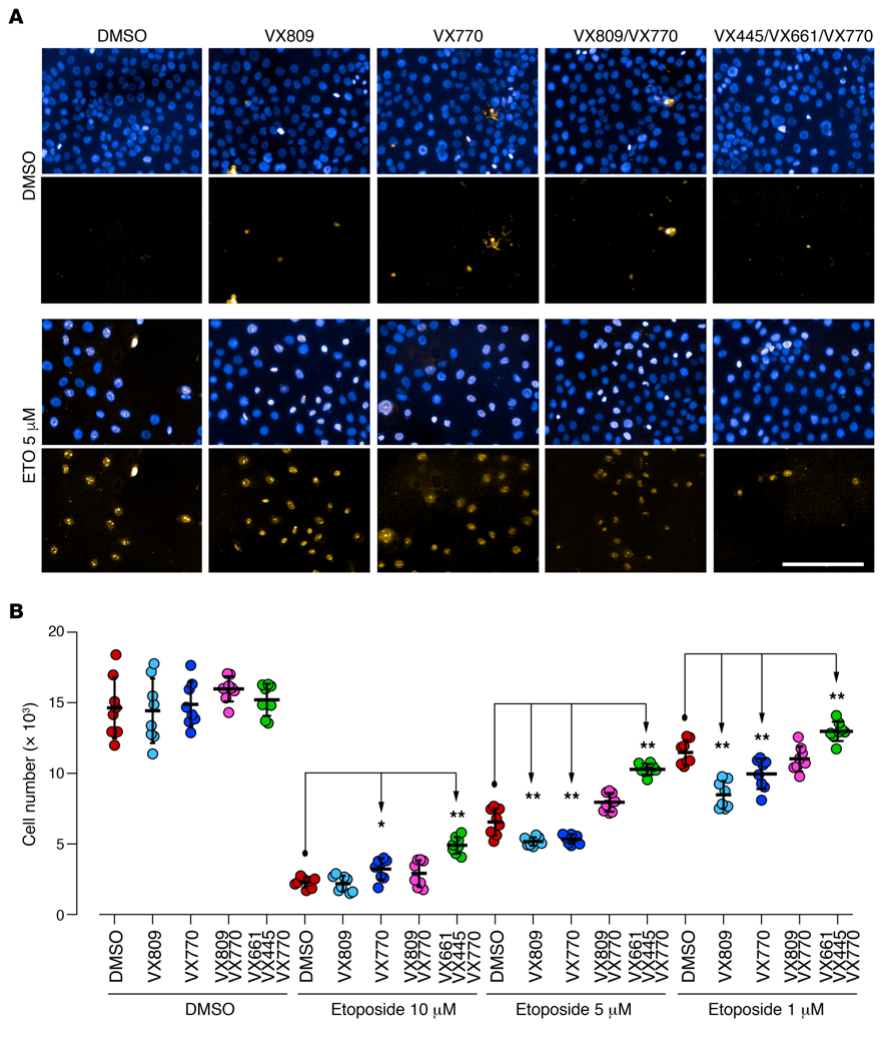
Susceptibility of bronchial cells treated with CFTR modulators to undergo apoptosis. CFBE41o- cells were treated for 24 hours with vehicle alone or with the indicated concentrations of etoposide to induce apoptosis, in the absence or presence of different CFTR modulators. Samples were then analyzed by automated high-content imaging and analysis. (**A**) Representative images. Scale bar: 200 μm. (**B**) Quantification of the number of viable CFBE41o- cells per condition. Data represent mean ± SEM, *n* = 5. *P < 0.05, **P < 0.01 compared with respective control (DMSO-treated) cells, 1-way ANOVA test with Dunnett’s post hoc test.

Given the great expectations associated with Trikafta for the management of CF, we focused our analysis on the binary comparison of the Trikafta group with the control group. The control and Trikafta groups are already separated by unsupervised PCA, as reported in Figure 5A, thus demonstrating that this drug alters the lipid profile of CFBE41o- in a clearly detectable way. Following a subsequent *t* test, with correction for multiple testing (FDR-adjusted *P* = 0.05), we outlined those features changing by at least 1.5-fold in a statistically significant way (see the corresponding volcano plot, Figure 5B). These lipids (37 downregulated and 22 upregulated with the treatment) are reported in Supplemental Table 2. This dual comparison highlights some other interesting changes in the molecular composition of the CFBE41o- lipidome. As reported in Supplemental Figure 9, a general downregulation of ceramides in the untargeted data set appeared to be associated with the CFTR rescue promoted by Trikafta. To confirm these data and to evaluate the ceramide levels in all the groups, we performed a second targeted LC-MS/MS experiment, focusing on 6 molecules [Cer(d18:1/16:0), Cer(d18:1/18:0), Cer(d18:1/20:0), Cer(d18:1/22:0), Cer(d18:1/24:1), Cer(d18:1/24:0)] (Figure 6), normally among the most abundant in mammalian tissues (39). This experiment showed that, while a trend for downregulation was observed also for VX-770 alone and VX-770/VX-890, the triple combination is the only treatment that lowers the levels of *all 6* major ceramides simultaneously and in a statistically significant way. To investigate the biological relevance of the decrease in ceramide expression in bronchial cells, we evaluated the susceptibility of CFBE41o- cells to undergo apoptosis. It was indeed demonstrated that ceramides act as second messengers involved in the induction of apoptosis (40, 41) and that augmented ceramides levels are associated with lung endothelial and epithelial cell apoptosis in murine models exposed to cigarette smoke (42, 43). In addition, XM462, an inhibitor of ceramide biosynthesis, promotes cell survival (44). Thus, we reasoned that, by lowering ceramide levels, the triple combination VX-661/VX-445/VX-770 might decrease the susceptibility of cells to undergo apoptosis following exposure to submaximal proapoptotic stimuli. To this aim, CFBE41o- cells expressing F508del-CFTR were plated at low density on high-quality 96-well plates suitable for imaging. After 24 hours, the cells were exposed to different concentrations (1–10 μM) of etoposide, a topoisomerase II inhibitor able to induce apoptosis in bronchial cells (45), in the absence or in the presence of single or combined CFTR modulators. The following day, by means of counterstaining with Hoechst 33342 and propidium iodide, we evaluated the number of living cells (i.e., cells negative for propidium iodide staining) for each condition (Figure 7A). None of the CFTR modulators (as single drugs or as combinations) affected the count of viable cells under resting conditions (i.e., in the absence of etoposide), demonstrating that CFTR modulators do not influence cell proliferation rate. On the contrary, treatment with etoposide alone caused a dose-dependent decrease in the number of viable cells (Figure 7B). However, when etoposide was added to the cells in the presence of the triple combination VX-661/VX-445/VX-770, we consistently observed a higher number of viable cells, as compared with wells treated with etoposide alone (Figure 7B). No other CFTR modulator or combination consistently affected cell counts. Taken together, these results demonstrate that the triple combination decreased cell susceptibility to undergo apoptosis following exposure to submaximal proapoptotic stimuli. This protective role might be associated with the downregulation of all 6 major ceramides observed following treatment with the triple combination (Figure 6).

**Figure 8:**
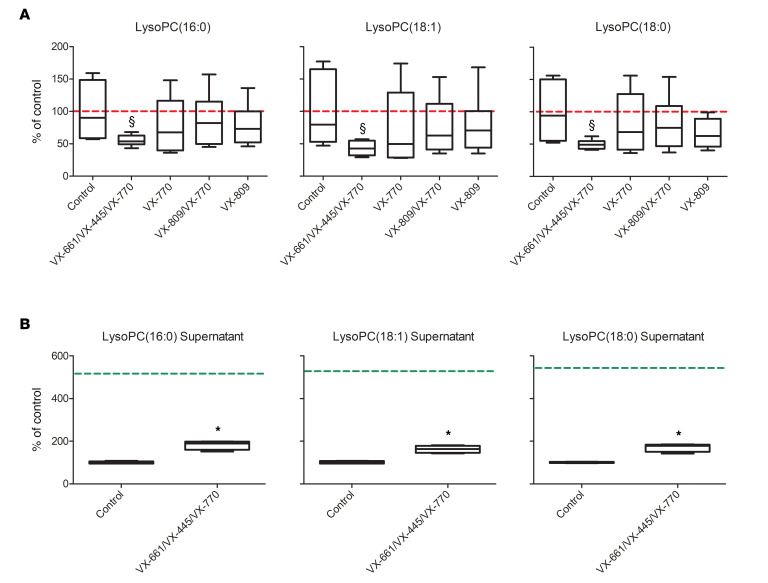
Targeted quantification LysoPCs in the CFBE41o- cells. (**A**) LysoPC levels in lysates following treatment with the triple combination (VX-661/VX-445/VX-770) after 24 hours of incubation. The average level of the control group is represented by the dotted red line. ^§^*P* < 0.05 compared with control, 1-way ANOVA test with Dunnett’s post hoc test. (**B**) LysoPC levels in the supernatants of the same CFBE41o- cells at 24 hours, following treatment with the triple combination. The average level in the medium at the beginning of the incubation is indicated by the dotted green line. Box-and-whiskers plot represent the median value (inner bar), the first and third quartile (box), and the minimum and maximum value (outer bars). Data represent mean ± SEM, *n* = 5. *P < 0.05 compared with control, 2-tailed *t* test.

Less clear is the rationale for the downregulation of the 3 major LysoPCs observed in the untargeted analysis (Supplemental Figure 9). Beside their reported importance as possible CF biomarkers in some biofluids (15, 46), little is known about the role of these molecules in CF physiology. Among the many biological functions in which LysoPC are involved, these molecules are known to be secreted to attract macrophages (47). In addition to their signaling role, however, LysoPCs are *also* synthetic precursors: they are condensed with acyl-CoA to produce PCs and glycerophosphocholine to regulate membrane fluidity in mammalian cells (48). To better investigate this point, as we did for ceramides, we selectively measured these 3 lipids in CFBE41o- cells by the means of a targeted LC-MS/MS experiment. While the decrease in *all 6* ceramides appeared to be associated with the treatment with Trikafta only, some of them were downregulated following treatment with VX-809/VX-770 (Figure 6). On the contrary, Figure 8A shows that the decrease of these LysoPCs was a *distinctive feature* of the treatment with the triple combination only. To gain a better sense of this point, we also measured the levels of LysoPC(16:0), LysoPC(16:1), and LysoPC(18:0) in the supernatant of the same CFBE41o- cells (triple combination versus control only). Figure 8B shows that the decrease in the CFBE41o- lipidome was associated with a *reduced consumption* of the same lipids from the incubation medium. This evidence allowed us to speculate that cells treated with the triple combination *simply use less* LysoPC from the medium to produce some of the PCs that are downregulated in triple combination cells (Supplemental Table 1).

## Discussion

Our work represents the first untargeted exploration of the changes in the global lipidomic profiles of the F508del-CFTR bronchial epithelial cells in response to CFTR rescue by drugs to our knowledge. The experiments were performed on independent cell cultures, established over a broad time frame; our results thus incorporate a realistic biological variability. We demonstrated that each of the tested drugs and each combination produces a characteristic set of changes in the cell lipidome that goes beyond the biological variability of different cell cultures. We showed that VX-770 treatment is associated with a particular lipid signature of 7 phosphatidylcholines and a ceramide. We then focused on the triple-drug combination (Trikafta), for which we observed a decrease of 6 ceramides. We confirmed this evidence in a second targeted experiment. This decrease in ceramide content is consistent with a beneficial effect of the drug caused *also* by a reduction of these proapoptotic stimuli, as demonstrated by a dedicated experiment on CFBE41o- cells. We also show that the treatment with this drug combination is associated with a decrease of 3 LysoPCs in the cell lipidome, with concomitant higher levels of these lipids in the supernatant. While the evidence is clear, a lot remains to be explained in the balance of LysoPC metabolism in CF. In this paper, we speculate that our data suggest a reduced use of these LysoPCs by Trikafta-treated cells that triggers a particular lipid membrane remodeling. We are well aware that our untargeted survey, albeit generic and unbiased, still does not cover the whole-cell lipidome. For many technical reasons, some lipid families are often underrepresented in these kinds of untargeted experiments. Future efforts might perhaps move on from our results to implement targeted profiling of individual lipid families. For example, phosphatidylinositols and their phosphates (PI and PIPx), very important signaling lipids known to be involved in CF (49), require dedicated sample preparation and analytical strategies and are thus virtually absent from our untargeted data set. It should also be noted that our findings might be only partially translated to primary cells. Indeed, the clear lipidomic alterations we observed in immortalized bronchial cells might be less evident in primary cells, in particular when compared with interindividual variability. Our findings should thus be followed by future larger-scale confirmatory studies. We nevertheless believe that our study will represent a helpful data set for future research. Looking ahead, if the crucial role of cell lipidome in CFTR rescue is confirmed, new scenarios will open up for the future of CF pharmacology. Strategies for lipidome remodeling might be used to enhance and support the pharmacological modulation of CFTR, thus ameliorating the CF phenotype and patient status. The active modulation of the cell membrane lipidome (membrane lipid therapy) has already been proposed for many different applications (50–52). Quite surprisingly, membrane lipid therapy has never been proposed for CF, perhaps because of a lack of knowledge of the specific lipidomic alterations induced by the disease. In this perspective, we are hereby sharing with the worldwide CF community this body of data and evidence, collected on the most widely used in vitro cell line used to our knowledge to investigate CF pharmacology.

## Methods

### Chemicals, reagents, instruments, and analytical standards.

2-Propanol (IPA) was purchased from VWR Chemicals, and acetonitrile, ammonium formate, formic acid, methanol, and chloroform used for sample preparation and LC-MS/MS analysis were purchased from MilliporeSigma (catalog 1000292500, 70221, 5.33001, 632546, and 650498, respectively). C17 Ceramide (d18:1/17:0) was purchased from Avanti Polar Lipids (Alabaster). UPLC/MS and MS/MS systems and columns were from Waters. VX-770, VX-809, and VX-661 were purchased from SelleckChem.

### Synthesis and characterization of VX-445.

VX-445 was synthesized following a modified procedure reported previously (patent WO 2019/018395 Al; https://patentscope.wipo.int/search/en/detail.jsf?docId=WO2019018395). Full details regarding synthesis and chemical characterization are provided in the Supplemental Information file.

### Cell cultures and incubations.

CFBE41o- 41o- cells overexpressing F508del-CFTR were cultured until confluence, harvested, and prepared for untargeted lipidomics. To address the above described scientific questions, each F508del culture was then treated with vehicle alone (DMSO) or the following single drugs or combinations: VX-809 (3 μM), VX-661 (10 μM), VX-770 (5 μM), VX-445 (3 μM), VX-809/VX-770, VX-661/VX-770, VX-445/VX-770, VX-661/VX-445, and VX-661/VX-445/VX-770. Six independent experiments were performed to ensure reproducibility. For each cell culture (5 million cells per condition), cells were harvested in PBS and pelleted. The concentrations of VX-661 and VX-445 were chosen according to Keating et al. (23). Although VX-809 is not part of the newly developed combination drugs, we decided to test it because of the reported negative effect on CFTR half-life at the plasma membrane following chronic coadministration of VX-770 and VX-809 (21, 22). CFTR rescue was verified as recently described (24).

### Evaluation of cell susceptibility to proapoptotic stimuli.

CFBE41o- cells stably expressing F508del-CFTR were plated at low density (10,000 cell/well) on 96-well plates suitable for high-content imaging. After 24 hours, cells were treated with test compounds at the desired concentrations or with vehicle alone (DMSO), in the absence or presence of different concentrations (1–10 μM range) of etoposide to induce cell apoptosis. The following day, plates were washed 3 times with D-PBS to remove dead cells and cell nuclei were counterstained with Hoechst 33342 and propidium iodide to visualized total and apoptotic cells, respectively. Plates were imaged with a ×10 air objective using the Opera Phenix (PerkinElmer) high-content screening system. The excitation of the Hoechst 33342 signal was at 405 nm, and the emission was at 435–480 nm. The propidium iodide signal was excited at 560 nm, and the emission was measured at 570–630 nm. Data are expressed as mean ± SEM, *n* = 8. Reproducibility of results was confirmed by performing 3 independent experiments. Statistical significance was tested by parametric ANOVA, followed by the Dunnett’s multiple comparisons test.

### Lipid extraction.

After trying the 3-phase Matyash (53) method, we observed that a simpler 2-phase extraction with isopropanol yielded very similar results (see Supplemental Figure 1) but with a dramatically reduced time and effort. This method has already been demonstrated to produce adequate and repeatable lipid coverage for untargeted experiments (54). Cell pellets were thus resuspended with H_2_O (50 μL), transferred to glass vials, and added with 1 mL isopropanol spiked with C17 Ceramide (1 μM) as internal standard. The samples were then vortexed for 10 minutes and sonicated for 10 minutes at room temperature. The samples were then centrifuged at 20,000 *g* for 20 minutes, and the supernatant (A) was carefully collected. Pellets were then reextracted with 100 μL of a mixture of methyl-tert-butyl ether/methanol (50:50, v/v), vortexed for 10 minutes, and sonicated for 10 minutes at room temperature. After further 20 minutes of centrifugation (20,000 *g*), the supernatant (B) was collected. Supernatants A and B were then pooled and dried under N_2_. At the time of analysis, the lipid extracts were dissolved in 200 μL methanol/chloroform (9:1, v/v) for untargeted LC-MS/MS analyses. All the samples, including procedure blanks, were extracted following this protocol. The whole sample set, consisting of a total of 64 samples, was then randomized, split into 4 batches, and analyzed by high-resolution LC-MS, using the methods already optimized by our group for untargeted lipidomics (24–27). For every batch, a QC sample was prepared by pooling together 5 μL of all the samples of that batch, including blanks and procedure blanks. For each batch, QC samples, consisting of pools of all the samples of the batch (including blanks) were prepared and analyzed together with the samples within each batch (6 injections: at the beginning, at the end, and in between the samples).

### Untargeted LC-MS/MS analysis.

The lipidomic analysis was carried out on a ACQUITY UPLC system coupled to a Synapt G2 QToF high-resolution mass spectrometer (Waters), acquiring both in positive (ESI^+^) and negative (ESI^−^) ion modes. Lipid separation was performed with a reverse-phase CSH C18 column (1.7 μm internal diameter; 2.1 × 50 mm, Waters). Eluents consisted of acetonitrile/water (60:40, v/v) with ammonium formate 10 μM (A) and isopropanol/acetonitrile (90:10, v/v) with ammonium formate 10 μM (B). Injection volume was 3 and 5 μL for ESI^+^ and ESI^–^, respectively. The flow rate was set on 0.450 mL/min, the column was kept at 50°C, and samples were eluted with the following gradient program: 0.0–1.0 minute, 10% B; 1.0–4.0 minutes, 10%–60% B; 4.0–8.0 minutes, 60%–75% B; 8.0–8.5 minutes 75%–100% B; 8.5–10.0 minutes, 100% B; and 10.0–10.1 back to 10% B. The column was then reconditioned for 1.9 minutes. The total run time was 12 minutes. Scan range was set from 50 to 1200 *m/z*. Cone voltage was set at 35 V. Source temperature was set to 90°C, desolvation gas and cone gas (N_2_) flows were set to 800 and 50 L/h respectively, desolvation temperature was set to 400°C. Data were acquired in MSE mode, alternating MS and MS/MS scans (55). The scan time was set to 0.3 seconds, low collision energy was set to 4 eV, and high collision energy was ramped from 25 to 45 eV. Leucine enkephalin (2 ng/mL) was infused as lock mass for real-time spectra recalibration. Masslynx software (Waters) was used for data acquisition.

### Targeted ceramide and LysoPC analysis.

Ceramides were also quantified in a targeted experiment by using a triple-quadrupole instrument (Xevo-TQMS, Waters) coupled to a ACQUITY UPLC (Waters) chromatographic system, using our previously published method (39). The 3 LysoPCs were quantified by using the same general LC-MS/MS method and adding the 3 corresponding MRM experiments (496**→**184, 524**→**184, and 52**→**184 for LysoPCs 16:0, 18:0, and 18:1, respectively). Collision energy was set to 25 eV and cone voltage was set to 30V.

### Data analysis.

The observed features for both ESI^+^ and ESI^–^ polarities were extracted from the RAW data, integrated, and realigned over all the runs by using Markerlynx software (Waters). The following parameters were used: mass range from 200 to 1200 *m/z*, retention time (RT) range from 1 to 10 minutes, minimum intensity of 4000 ion counts, *m/z* tolerance for extracted ion current 0.03 *m/z*, and RT tolerance 0.03 minutes. Background ions observed in procedure blank and blank runs above the same threshold were automatically excluded by the software (background exclusion list). Exogenous d18:1/17:0 ceramide was set as internal standard, observed as [M-H_2_O+H]^+^ and [M+HCOO]^–^ adducts for ESI^+^ and ESI^–^ ion modes, respectively, with RT 5.47 ± 0.03 minutes. Although cell pellets were washed with PBS before extraction, the *m/z* values of the drugs were excluded from the peak picking process. All the samples from all the 4 acquisition batches were realigned using this ceramide as a reference. The obtained data sets for ESI^+^ and ESI^–^ were then merged in a final data matrix (1571 features × 42 samples) and analyzed using Metaboanalyst (56) web-based software. After passing the data integrity check, missing values (empty cells) in the data were automatically replaced with the minimum value of each given feature in the corresponding column. The data set was then normalized by the sum of all the observed features, log transformed, and Pareto scaled (57). The absence of instrumental response drift in the data was then verified by checking the clustering of the QC samples within each batch (see Supplemental Figure 2). As reported in the text, both PCA and PLS-DA analyses were then performed. Cross-validation of PLS-DA analysis was performed by using the leave-one-out algorithm (26), searching over 5 principal components. Q2 value was used as a benchmark of potential overfitting. VIP score for PLS-DA was calculated over component 1. Cluster analysis was done using heatmaps by applying the Euclidean distance measurement and Ward algorithm (56) to normalized data. Correlation analysis among the features was performed using Pearson r as distance measure (58). Pathway analysis was performed on annotated data, using their HMDB (59) identifier. Hypergeometric test was used for overrepresentation analysis, and relative-betweeness centrality was used for pathway topology analysis on the KEGG (60) pathway library for homo sapiens.

### Feature annotation.

The relevant features, provisionally indicated by a RT_*m/z* value and selected from VIP scores and *t* tests from volcano plot analysis, were manually annotated. The corresponding *m/z* value at the given RT was first manually extracted from the RAW data. The observed accurate mass value was then searched against the Lipid Maps database (27). The following adduct species were searched: [M+H]^+^, [M-H_2_O+H]^+^, [M+NH_4_]^+^, [M+Na]^+^ for ESI^+^ and [M-H]^–^, [M+HCOO]^–^ for ESI^–^, allowing 0.01 *m/z* as maximum allowed tolerance for both polarities. The resulting list of proposed matches was then manually scrutinized, considering the consistency of the proposed structures with the observed RT (based on the well-known chromatographic behavior of lipids in reversed-phase mode, ref. 61) and with the observed MS/MS spectrum obtained in MSe mode for the given feature. None of the proposed annotations was confirmed with the use of an authentic analytical reference standard, so, based on current guidelines for reporting data in metabolomics (62, 63), the identification level of the lipids under investigation should be considered higher than 1. Lipid IDs should thus be considered putative throughout the text. For most of the proposed structures, we prefer not to make inferences on the exact composition of the fatty acyl chains and we thus report the condensed structure, for example DG(34:1) instead of DG(16:0/18:1).

### Data availability.

All the RAW data files related to this study are publicly available through the Metabolights (64) database. Raw files have been deposited in the EMBL-EBI MetaboLights database, with the identifier MTBLS1480. The complete data set can be accessed here: https://www.ebi.ac.uk/metabolights/MTBLS1480

### Statistics.

For binary comparisons, volcano plot analysis was used to select features based on their observed fold change between 2 groups (≥1.5) and their FDR-adjusted *P* value based on a 2-tailed *t* test (*P* < 0.05). For univariate data analysis, both 2-tailed unpaired *t* test and 1-way ANOVA were performed using GraphPad Prism (GLS Biotech). Dunnett’s post hoc test was used to compare all groups with each other.

### Study approval.

This study was performed on immortalized human cell lines; therefore, it does not need approval from an ethical committee. All the experiments were performed in accordance with common guidelines for cell cultures.

## Author contributions

NL, EP, CB, and SMB performed all the experimental activities; AG synthesized VX-445; TB contributed to the data interpretation; NP supervised the biological assays; and AA conceived the project, supervised the work, and wrote the manuscript with the help of NP.

## Supplementary Material

Supplemental data

Supplemental Table 1

Supplemental Table 2

Supplemental Table 3

## Figures and Tables

**Table 1:**
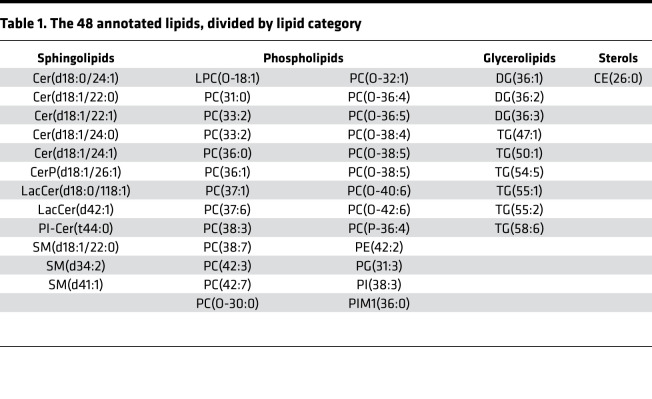
The 48 annotated lipids, divided by lipid category

## Version 1 07/16/2020

In-Press Preview

## Version 2 08/20/2020

Electronic publication
