# Supplementary material for: Distinctive lipid signatures of bronchial epithelial cells associated with cystic fibrosis drugs, including Trikafta
Source: JCI Insight. 2020 Aug 20;5(16):e138722. doi: 10.1172/jci.insight.138722 (PMC7455125; doi:10.1172/jci.insight.138722)
Supplement: Supplemental data [file jciinsight-5-138722-s117.pdf]

DISTINCTIVE LIPID SIGNATURES OF HUMAN BRONCHIAL EPITHELIAL CELLS ASSOCIATED WITH TREATMENT WITH CYSTIC FIBROSIS DRUGS, INCLUDING THE NEWLY APPROVED TRIPLE COMBINATION (TRIKAFTA®)

Nara Liessi[1§], Emanuela Pesce[2§], Clarissa Braccia[3], Sine Mandrup Bertozzi[1], Alessandro Giraudo[3], Tiziano Bandiera[3], Nicoletta Pedemonte[2,#] and Andrea Armirotti[1,#]

1) Analytical Chemistry Lab, Istituto Italiano di Tecnologia, Via Morego 30, 16163, Genova, Italy; 2) U.O.C. Genetica Medica, Istituto Giannina Gaslini, Via Gerolamo Gaslini 5, 16147 Genova, Italy; 3) D3PharmaChemistry, Istituto Italiano di Tecnologia, Via Morego 30, 16163, Genova, Italy

Supplementary Figures and Tables

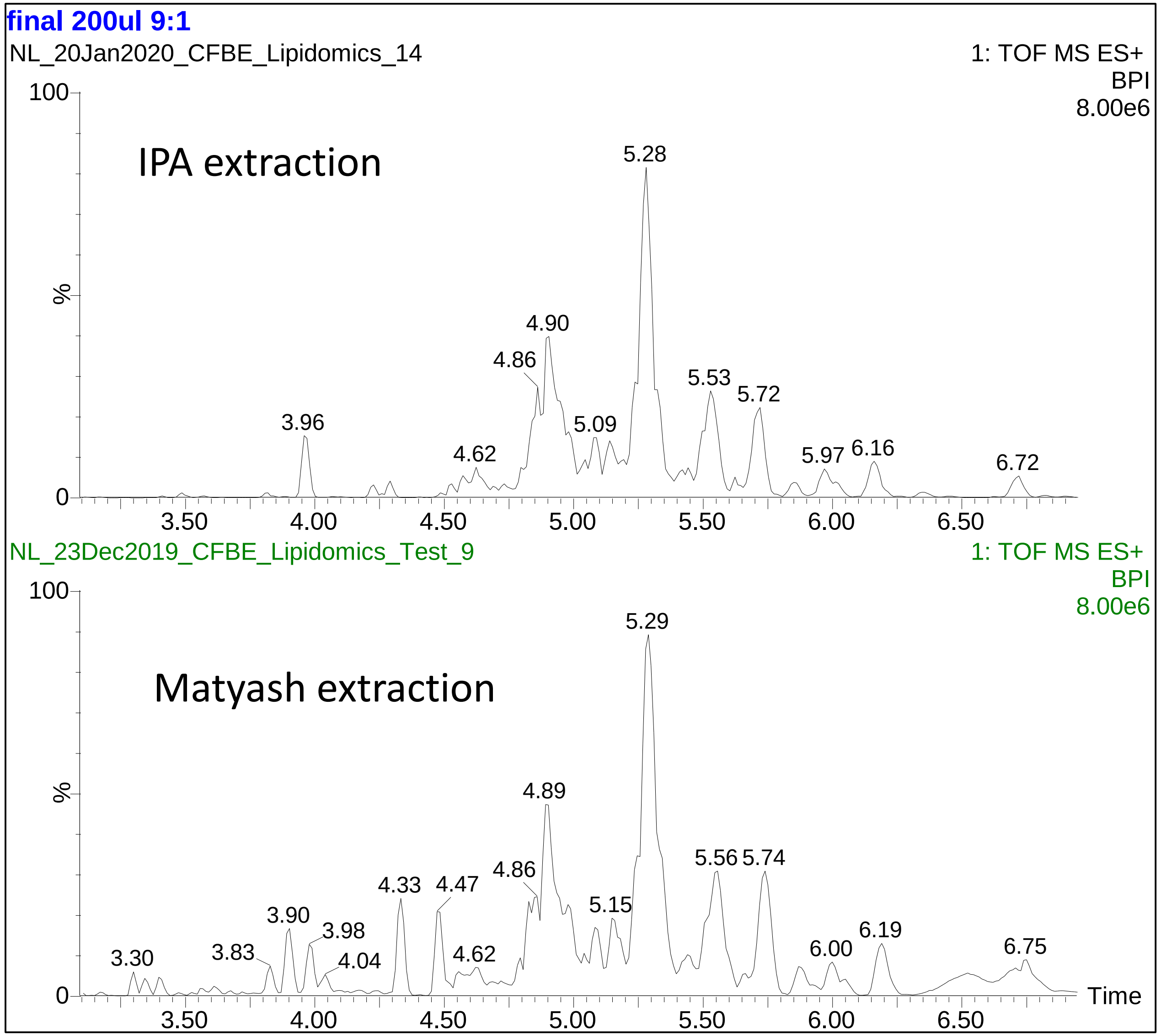

Figure S1: Comparable lipid recovery from CFBE cells (two independent preparations) using Matyash protocol (bottom) and extraction with isopropanol (top).

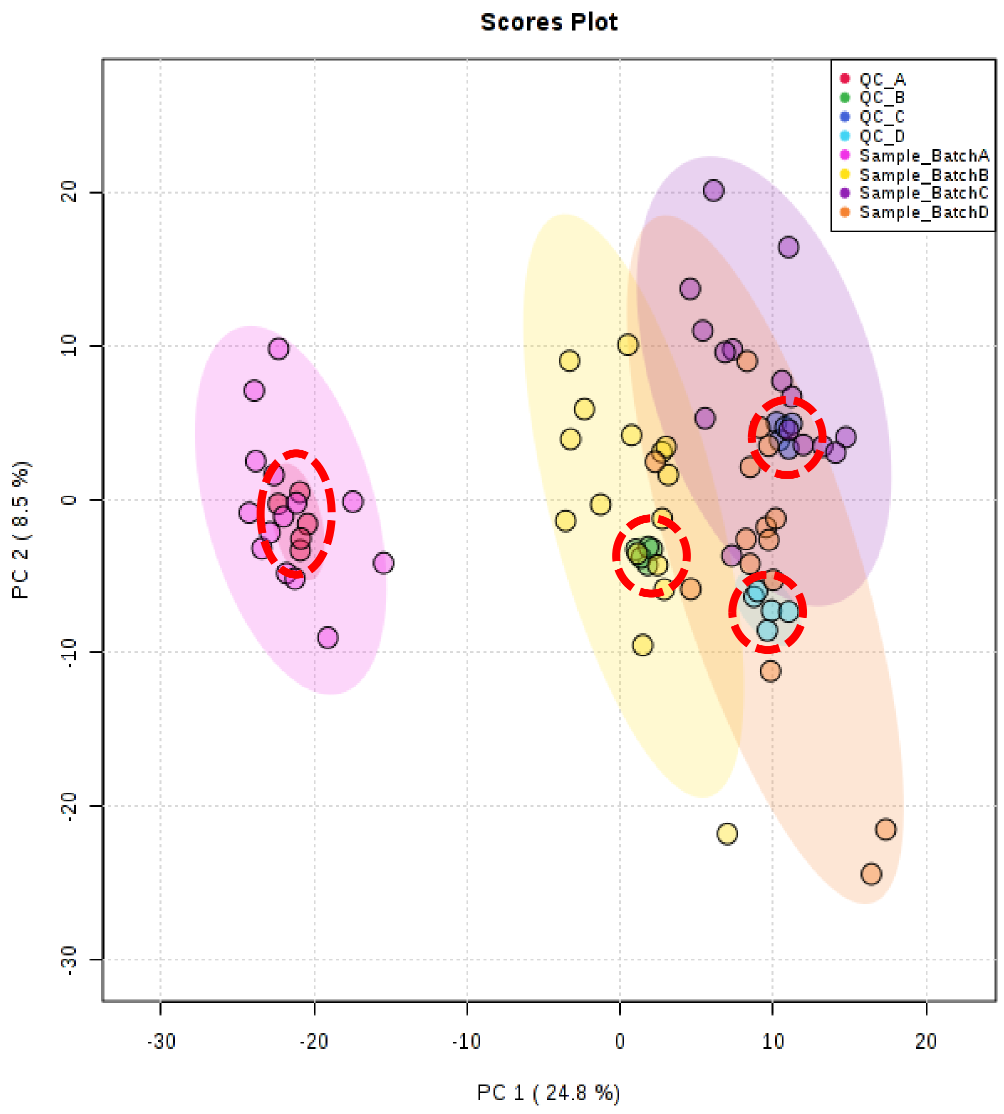

Figure S2: PCA Scores Plot of the four batches

- 7 Experimental Groups (6 independent cell preparations)
- 6 Procedure blank samples added
- 25 blank runs intermixed within the samples
- Total of 97 samples, randomized then splitted into 4 batches (23-25-25-24 samples)
- 1 QC group per batch (pool of samples of that batch, including blanks)
- Clusterization of QC samples is indicated by the red circle

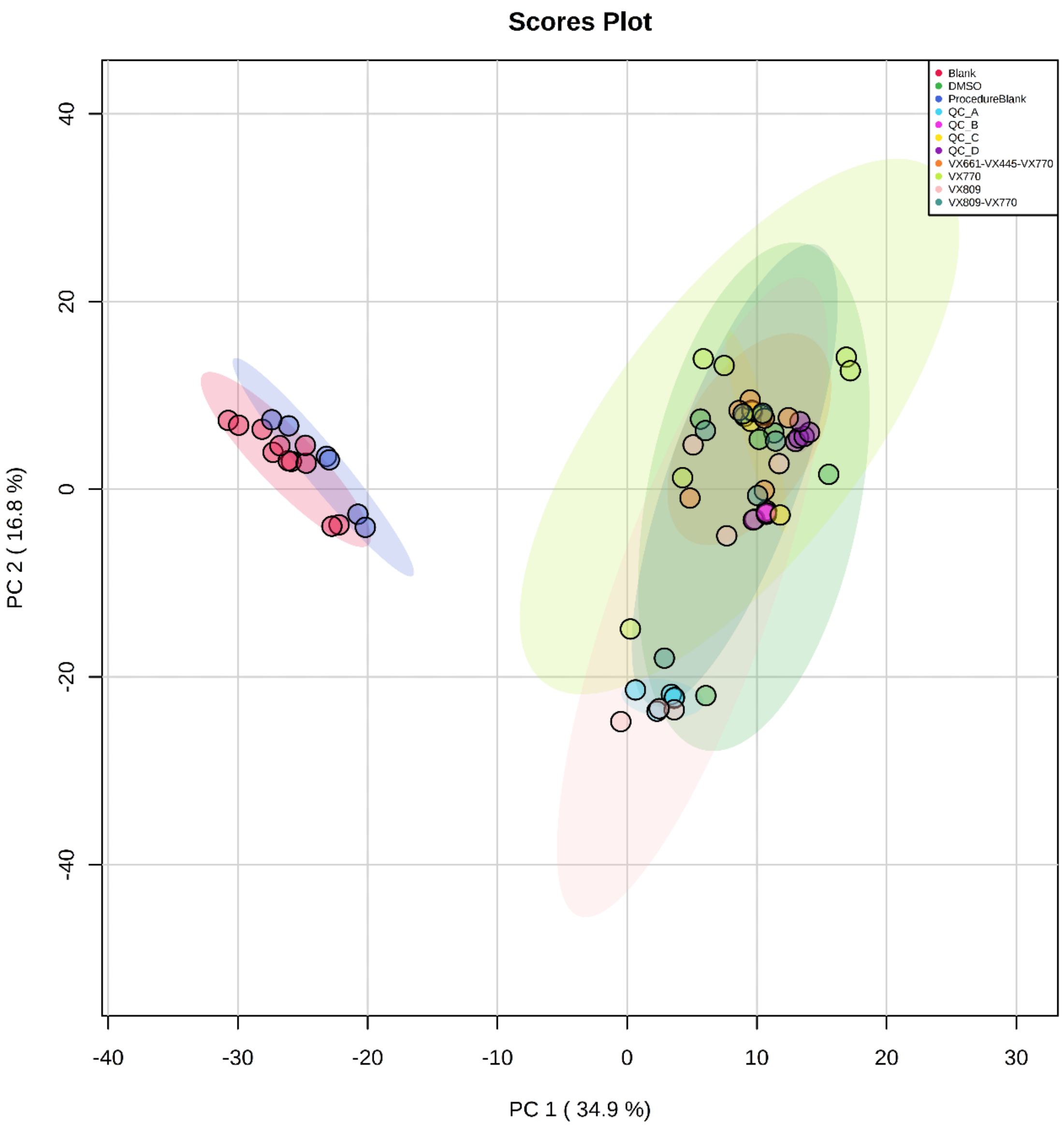

Figure S3: PCA Score Plot of all the features observed from all the experimental groups

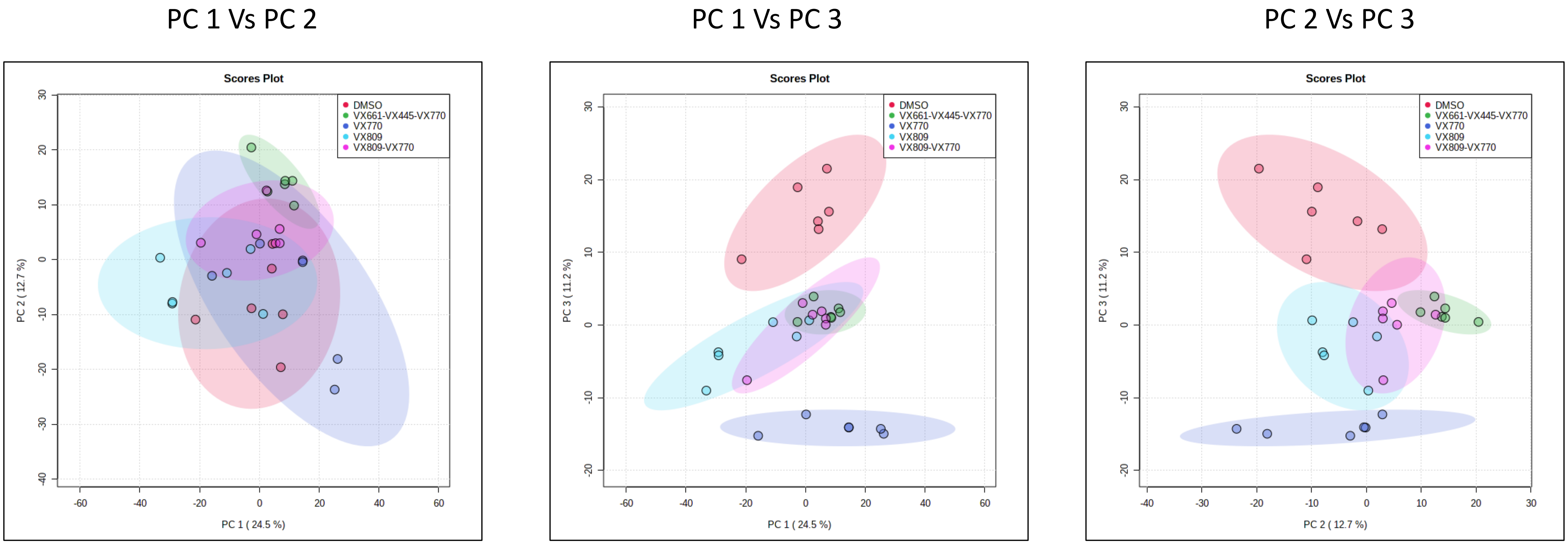

Figure S4: PCA Score Plot of all the features observed from the five experimental groups. The principal components used for the plot are indicated.

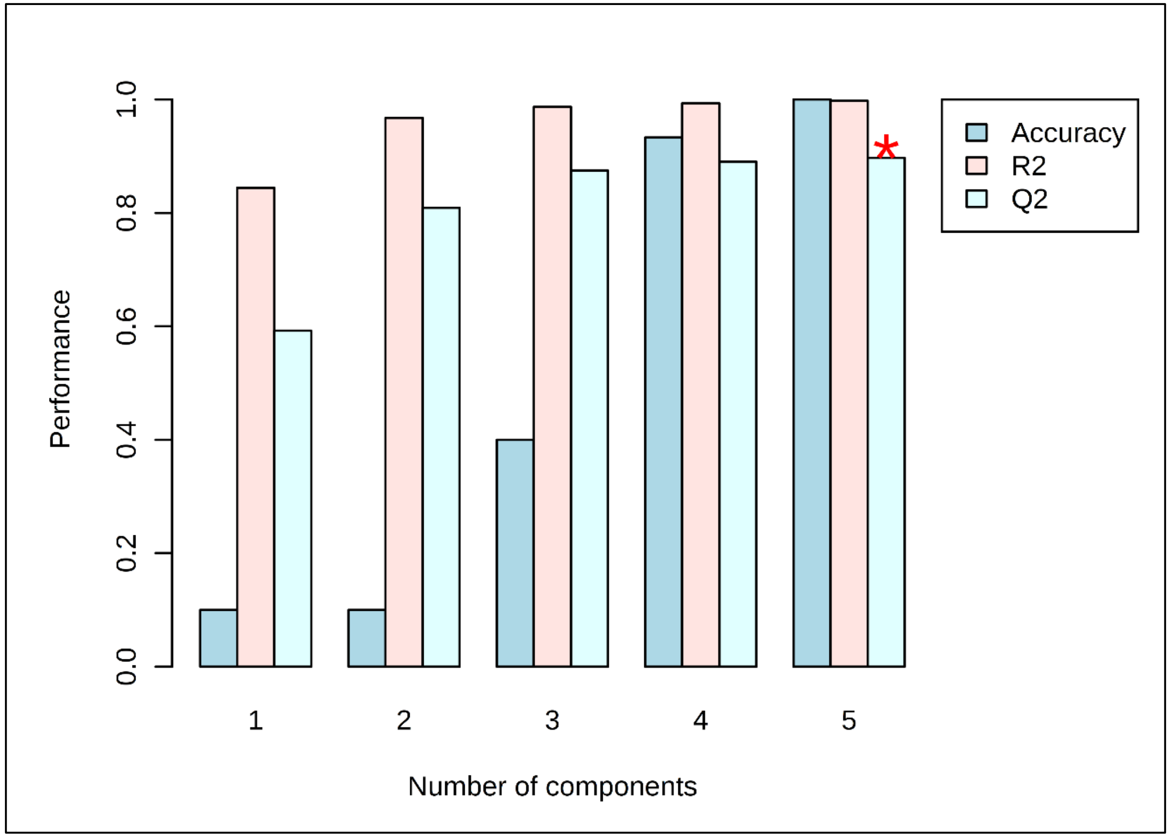

Figure S5: Results of leave-one-out cross validation of the PLS-DA model. Positive Q2 values (0.6 and 0.8 for 1 and 2 principal component respectively) indicate that the model is not overfitted.

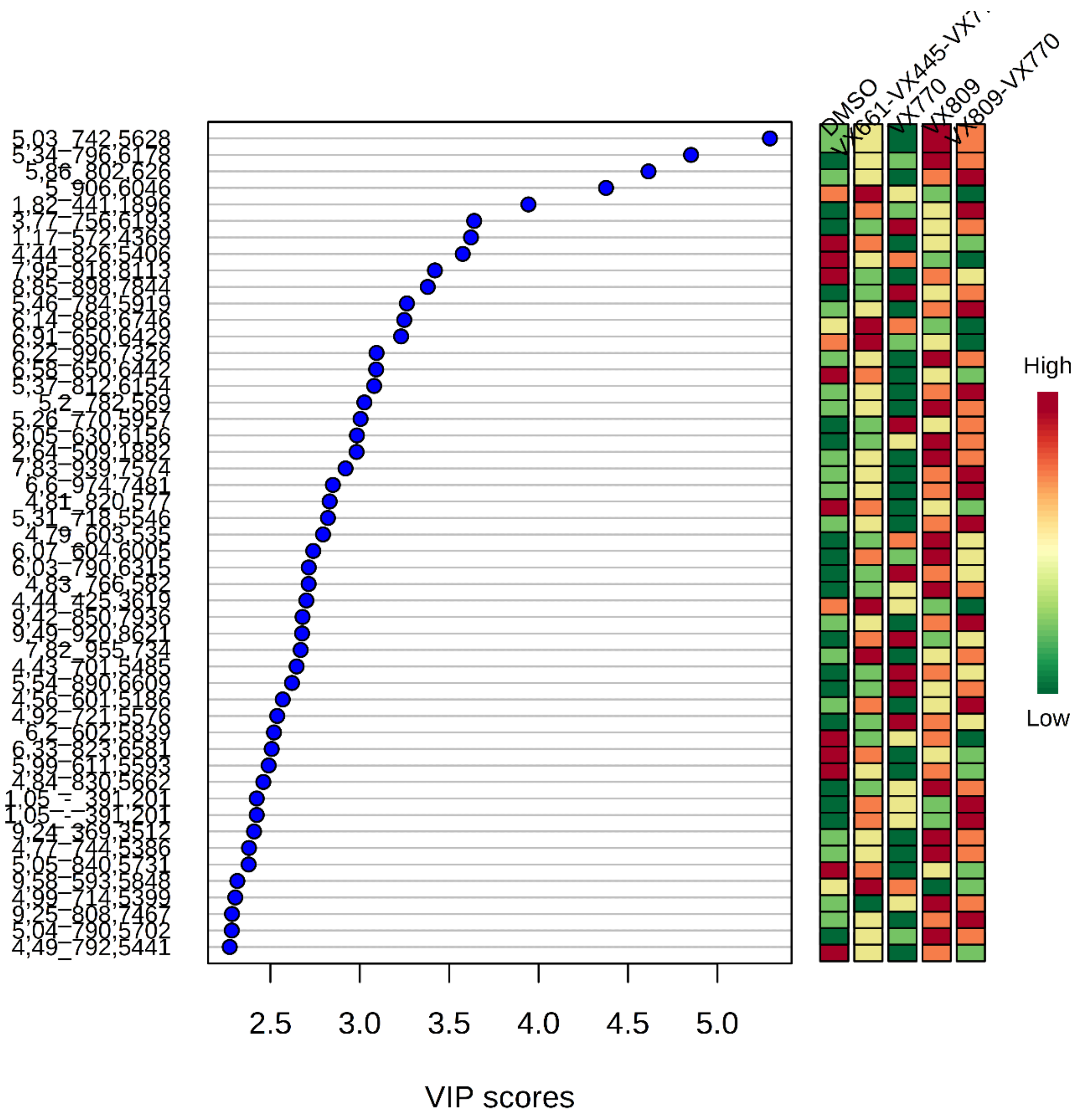

Figure S6: VIP Scores for PLS-DA analysis of CFBE cells lipidome treated with drugs or control DMSO. For each feature (retention time_m/z value), the relative abundance is reported for each group. The first 50 features are visualized here

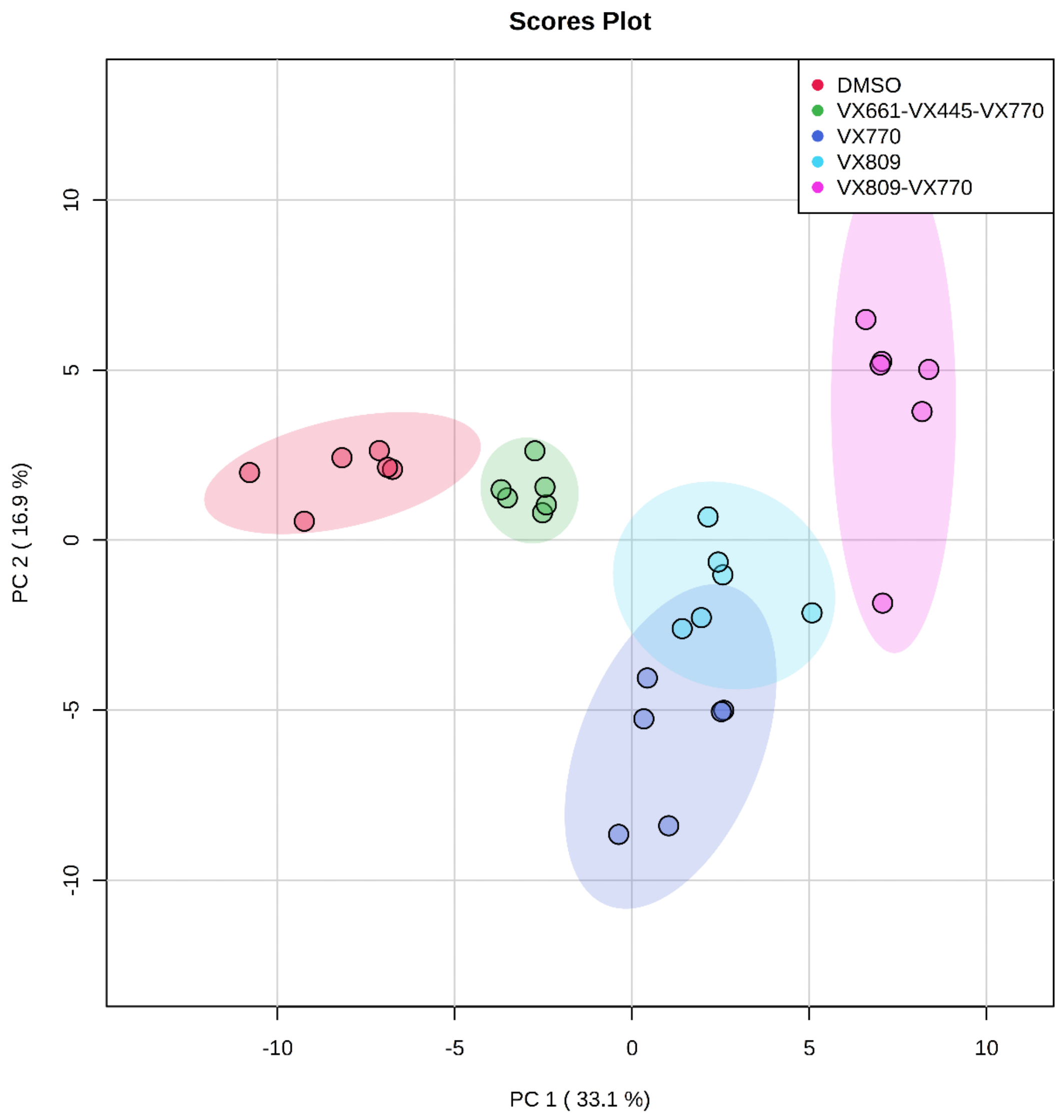

Figure S7: PCA Score Plot built with the 48 annotated features deriving from PLS-DA VIP list.

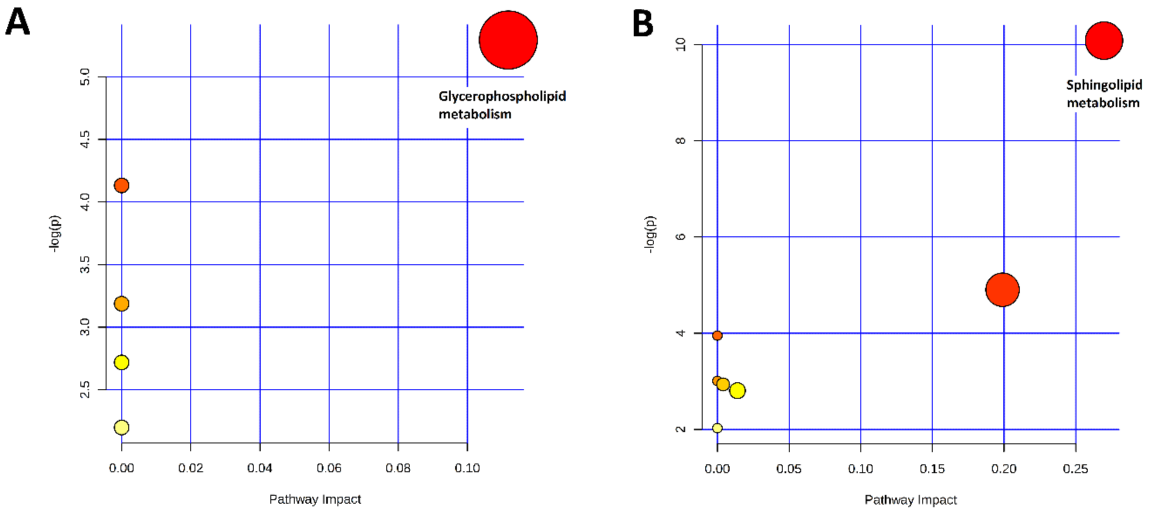

Figure S8: Pathway analysis for the two clusters observed in the correlation analysis of the 48 annotated lipids in the experimental groups. Glycerophospholipid metabolism is mostly enriched in Cluster 1 (Panel A); sphingolipid metabolism in Cluster 2 (Panel B).

	Average FC Vs control (downregulation)		
**Putative Lipid ID**	**VX-445/661/770**	**VX-770**	**VX-809/770**
Cer(d18:1/16:0)	2.26	1.96	1.92
PC(32:2)	1.76	1.78	1.52
PC(36:5)	2.26	1.89	1.63
PC(38:7)	1.84	2.18	1.65
PC(42:10)	1.72	4.94	2.96
PC(P-36:4)	1.89	2.08	1.95
PC(P-36:5)	1.90	2.54	3.05
PC(P-38:6)	2.26	2.79	2.35

Table S1: 8 lipid species downregulated in all VX-770 groups.

ID	Class	FC	P-value
Cer(d18:1/16:0)	Ceramide	2.2554	0.006237
Cer(d18:1/18:0)	Ceramide	2.195	0.020879
Cer(d18:1/20:0)	Ceramide	2.9017	0.020337
Cer(d18:1/22:0)	Ceramide	2.4049	0.027552
Cer(d18:1/22:1)	Ceramide	2.9989	0.010648
Cer(d18:1/23:0)	Ceramide	2.6228	0.014493
Cer(d18:1/24:0)	Ceramide	1.6589	0.043204
Cer(d18:1/24:1)	Ceramide	2.37	0.034133
HexCer(d18:1/16:0)	Ceramide	1.8749	0.005312
DG(32:0)	Diacylglycerol	2.0746	0.000426
DG(32:1)	Diacylglycerol	2.2496	0.049531
DG(34:1)	Diacylglycerol	1.6163	0.045091
DG(34:2)	Diacylglycerol	1.6895	0.003433
DG(38:4)	Diacylglycerol	2.1839	0.00296
DG(38:6)	Diacylglycerol	2.1133	0.028234
DG(O-34:1)	Diacylglycerol	2.0957	0.030244
LysoPC(16:0)	Lysophosphatidylcholine	1.8371	0.022976
LysoPC(18:0)	Lysophosphatidylcholine	1.6363	0.032615
LysoPC(18:1)	Lysophosphatidylcholine	2.3612	0.037291
PC(32:2)	Phosphatidylcholine	1.759	3.77E-05
PC(34:4)	Phosphatidylcholine	1.5838	0.008845
PC(36:4)	Phosphatidylcholine	1.5373	0.000494
PC(36:5)	Phosphatidylcholine	2.2619	0.000306
PC(36:6)	Phosphatidylcholine	1.8339	0.00171
PC(37:6)	Phosphatidylcholine	2.4602	0.026803
PC(38:7)	Phosphatidylcholine	1.8418	0.002183
PC(42:10)	Phosphatidylcholine	1.7173	0.048486
PC(P-30:0)	Phosphatidylcholine	1.9132	0.016953
PC(P-36:4)	Phosphatidylcholine	1.886	0.001822
PC(P-36:5)	Phosphatidylcholine	1.9008	0.023852
PC(P-38:6)	Phosphatidylcholine	2.259	0.017276
PE(36:5)	Phosphatidylethanolamine	4.3202	0.000972
PE(40:5)	Phosphatidylethanolamine	1.5512	0.007548
PE(P-38:6)	Phosphatidylethanolamine	2.2888	0.008116
PS(40:6)	Phosphatidylserine	1.5643	0.017872
SM(d18:1/14:0)	Sphingomyelin	1.8875	0.03763
SM(d18:1/15:0)	Sphingomyelin	2.7142	0.031264

Downregulated with triple combination

ID	Class	FC	P-value
Cer(d18:0/24:0)	Ceramide	0.50994	0.031223
LacCer(d18:1/14:0)	Ceramide	0.59577	0.003834
Cer(m18:0/18:0)	Deoxyceramide	0.57443	0.004351
Cer(m18:0/24:0)	Deoxyceramide	0.52914	0.035616
DG(38:2)	Diacylglycerol	0.63285	0.006117
DG(42:7)	Diacylglycerol	0.63904	5.00E-05
DG(44:7)	Diacylglycerol	0.63033	0.006567
PC(42:1)	Phosphatidylcholine	0.53134	0.014274
PC(44:1)	Phosphatidylcholine	0.37005	0.033365
PC(P-32:0)	Phosphatidylcholine	0.63285	3.38E-05
PC(P-32:1)	Phosphatidylcholine	0.54456	0.04927
PC(P-40:3)	Phosphatidylcholine	0.44135	0.003321
PC(P-40:4)	Phosphatidylcholine	0.49386	9.59E-05
PC(P-40:5)	Phosphatidylcholine	0.63025	3.07E-05
PC(P-40:6)	Phosphatidylcholine	0.5771	0.003113
PE(36:1)	Phosphatidylethanolamine	0.61834	0.023646
PE(P-33:0)	Phosphatidylethanolamine	0.61089	2.56E-05
PE(P-35:1)	Phosphatidylethanolamine	0.6073	0.000751
SM(d18:0/22:0)	Sphingomyelin	0.53482	0.004552
SM(d18:0/24:0)	Sphingomyelin	0.41493	0.002416
SM(d18:1/24:0)	Sphingomyelin	0.53277	0.002383
TG(56:4)	Triacylglycerol	0.57384	0.038547

Upregulated with triple combination

Table S2: Significantly up and downregulated lipid species in triple combination group compared to control (Fold change >1.5, p-value <0.05 in 2 tails t-test)

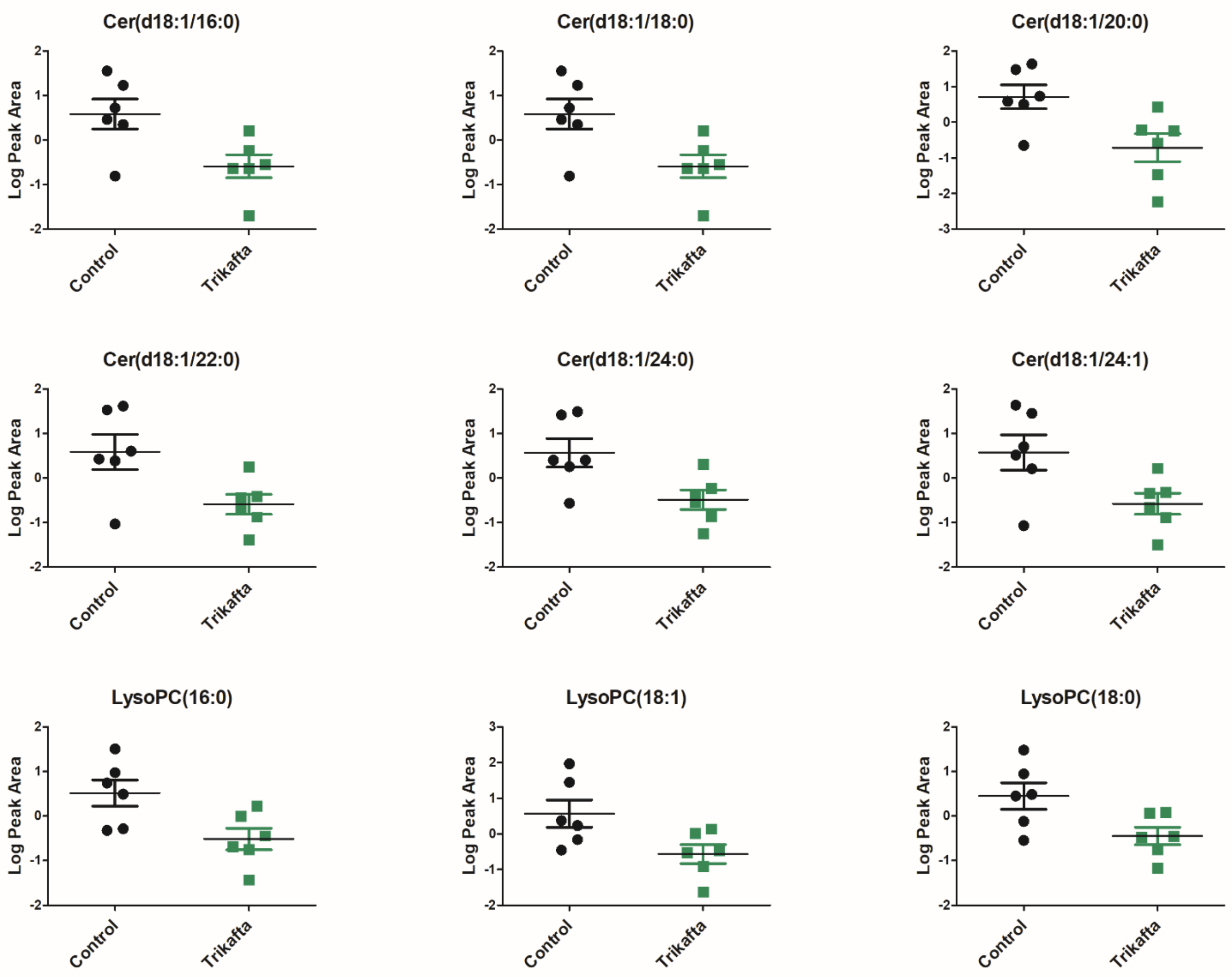

Figure S9: Results of a t-test comparison between control and Trikafta® groups done using the untargeted dataset. All the changes reported in the plot have a p-value ranging from 0.019 to 0.035.

SUPPLEMENTARY INFORMATION: SYNTHESIS OF VX-445

Compound **VX-445** was synthesized following a modified procedure reported by Haseltine, E. L. *et al.* (WO 2019/018395 Al).

Solvents and reagents were obtained from commercial suppliers and were used without further purification. Automated column chromatography purifications were performed on Teledyne ISCO apparatus (CombiFlash® Rf) with pre-packed silica gel columns of different sizes (Redisep). NMR experiments were run on a Bruker Avance III 400 system (400.13 MHz for ^{1}H, and 100.62 MHz for ^{13}C), equipped with a BBI probe and Z-gradients and Bruker FT NMR Avance III 600 MHz spectrometer equipped with a 5 mm CryoProbeTM QCI ^{1}H/^{19}F–^{13}C/^{15}N–D quadruple resonance, a shielded z-gradient coil and the automatic sample changer SampleJet™ NMR system (600 MHz for ^{1}H, 151 MHz for ^{13}C and 565 MHz for ^{19}F). Chemical shifts for ^{1}H and ^{13}C spectra were recorded in parts per million using the residual non-deuterated solvent as the internal standard (for $CDCl_3$: 7.26 ppm, ^{1}H and 77.16 ppm, ^{13}C; for DMSO-d_6: 2.50 ppm, ^{1}H; 39.52 ppm, ^{13}C; for D_2O: 4.79 ppm, ^{1}H). The specific rotation was measured by using an Autopol II automatic polarimeter (Rudolph Research Analytical). The analyses by UPLC/MS were run on a Waters ACQUITY UPLC/MS system consisting of a SQD (Single Quadrupole Detector) Mass Spectrometer equipped with an Electrospray Ionization interface and a Photodiode Array Detector. The PDA range was 210-400nm. Electrospray ionization in positive and negative mode was applied in the mass scan range 100-650Da or 150-750Da. The analyses were performed on either an ACQUITY UPLC HSS T3 C_{18} column (50x2.1mmID, particle size 1.8µm) with a VanGuard HSS T3 C_{18} pre-column (5x2.1mmID, particle size 1.8µm) (LogD<1: *Polar method*) or an ACQUITY UPLC BEH C_{18} column (50x2.1mmID, particle size 1.7µm) with a VanGuard BEH C_{18} pre-column (5x2.1mmID, particle size 1.7µm) (LogD>1: *Generic and Apolar methods*). The mobile phase was 10mM NH_4OAc in H_2O at pH 5 adjusted with AcOH (A) and 10mM NH_4OAc in MeCN-H_2O (95:5) at pH 5 (B) with 0.5mL/min as flow rate. Different linear gradients were applied depending on LogD of the compounds. *Polar method (LogD<1)*: 0-0.2min: 0%B, 0.2-2.7min: 0-50%B, 2.7-2.8min: 50-100%B, 2.8-3.0min: 100%B; *Generic method (LogD>1)*: 0-0.2 min: 5%B, 0.2-2.7 min: 5-95%B, 2.7-2.8 min: 95-100%B, 2.8-3.0 min: 100%B; *Apolar method (LogD>1)*: 0-0.2 min: 50%B, 0.2-2.7 min: 50-100%B, 2.7-3.0 min: 100%B.

Compounds (*R*)-**6** and (*S*)-**6** were obtained by semi-preparative chiral HPLC on a Waters HPLC instrument consisting of a 1525 Binary HPLC Pump, 2998 Photodiode Array Detector and a

Waters Fraction Collector III. The separation was performed on a Daicel ChiralCel ODH column (250×10mmID, particle size 5µm) using Heptane/EtOH (95:5 v/v) as mobile phase at a flow rate of 5 mL/min. Determination of enantiomeric excess (ee) for compounds (*R*)-**6** and (*S*)-**6** was performed on a Waters Alliance HPLC instrument consisting of an e2695 Separation Module and a 2998 Photodiode Array Detector using a Daicel ChiralCel ODH column (250×4.6 mmID, particle size 5µm) and Heptane/EtOH (95:5 v/v) as mobile phase with a flow rate of 1 mL/min.

Determination of enantiomeric excess (ee) for **VX-445** was performed on the HPLC system described above using a Daicel ChiralPak AD column (250×4.6 mmID, particle size 10µm) and Heptane/EtOH (95:5 v/v) as mobile phase at a flow rate of 1 mL/min.

The absolute configuration of **VX-445** was determined by comparison of $[\alpha]_D$ and chiral HPLC t_R with a standard compound purchased from Selleck Chemicals LLC.

Synthesis of intermediate **4**.

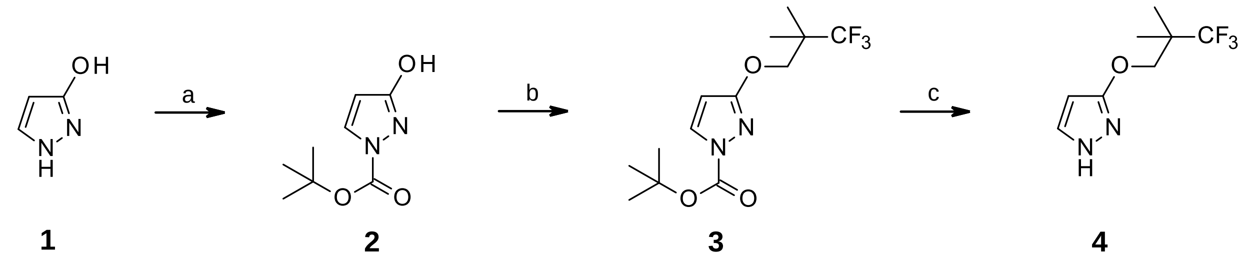

Scheme 1. Reagents and conditions: (a) Boc anhydride, Et_3N, CH_2Cl_2, rt; (b) 3,3,3-Trifluoro-2,2-dimethyl-propan-1-ol, PPh_3, DIAD, toluene, 110 °C; (c) 4M HCl in dioxane, 45 °C.

***tert*-Butyl 3-hydroxypyrazole-1-carboxylate (2).** To a solution of 1H-pyrazol-3-ol (**1**, 0.50 g, 5.95 mmol) in anhydrous CH_2Cl_2 (5.0 mL) were added di-*tert*-butyl dicarbonate (1.43 g, 6.54 mmol) and triethylamine (0.91 mL, 6.54 mmol). The resulting mixture was stirred at rt for 24 h. The solution was diluted with CH_2Cl_2 (20 mL), NH_4Cl saturated solution (20 mL) was added and the biphasic solution was partitioned. The water phase was extracted with CH_2Cl_2 (2 × 20 mL), and EtOAc (2 × 20 mL). The combined organic phase was dried over Na_2SO_4 and concentrated. Purification by flash chromatography (CH_2Cl_2/MeOH, 99:1 v/v) afforded **2** (0.656 g, 60%) as off-white solid. ^1H NMR (400 MHz, DMSO-d_6) δ 10.90 (s, 1H), 7.96 (d, J = 2.9 Hz, 1H), 5.88 (d, J = 2.9 Hz, 1H), 1.53 (s, 9H). UPLC-MS: t_R = 1.47 min (Generic method). MS (ESI) m/z calcd for $C_8H_{11}N_2O_3$ $[M-1]^-$: 183.1, found: 183.1.

***tert*-Butyl 3-(3,3,3-trifluoro-2,2-dimethyl-propoxy)pyrazole-1-carboxylate (3).** To a solution of **2** (0.292 g, 1.58 mmol), and 3,3,3-trifluoro-2,2-dimethyl-propan-1-ol (0.225 g, 1.58 mmol) in anhydrous toluene (9.0 mL) was added triphenylphosphine (0.457 g, 1.74 mmol) followed by diisopropyl azodicarboxylate (0.343 mL, 1.74 mmol). The resulting mixture was stirred at 110 °C for 24 h. The solution was evaporated, heptane (10 mL) was added followed by heptane/toluene 4:1 (10 mL). The insoluble was removed by filtration, washed with heptane/toluene 4:1 v/v (4 × 10 mL) and the combined filtrate was evaporated. Purification of the residue by flash chromatography (cyclohexane/EtOAc, 95:5 v/v) afforded **3** (0.305 g, 62%) as white solid. ^1H NMR (400 MHz, $CDCl_3$) δ 7.84 (d, J = 2.9 Hz, 1H), 5.89 (d, J = 2.9 Hz, 1H), 4.26 (s, 2H), 1.62 (s, 9H), 1.23 (s, 6H). UPLC-MS: t_R = 1.57 min (Apolar method). MS (ESI) m/z calcd for $C_{13}H_{20}F_3N_2O_3$ $[M+1]^+$: 309.1, found: 309.1.

3-(3,3,3-Trifluoro-2,2-dimethyl-propoxy)-1H-pyrazole (4). Compound **3** (0.61 g, 1.97 mmol) was dissolved in 4 M HCl in dioxane (5.0 mL) and stirred at 45 °C. After 2.5 h the solution was evaporated and the residue was taken in EtOAc (50 mL) and H_2O (50 mL). The layers were partitioned and the water phase was extracted with EtOAc (2 × 50 mL). The combined organic phase was washed with brine, dried over Na_2SO_4, and evaporated to give **4** (0.41 g, 99%) as white solid. ^1H NMR (400 MHz, $CDCl_3$) δ 7.37 (d, J = 2.5 Hz, 1H), 5.76 (d, J = 2.5 Hz, 1H), 4.13 (s, 2H), 1.26 (s, 6H). UPLC-MS: t_R = 0.60 min (Apolar method). MS (ESI) m/z calcd for $C_8H_{12}F_3N_2O$ $[M+1]^+$: 209.1, found: 209.0.

Synthesis of intermediate (***S***)-**7**.

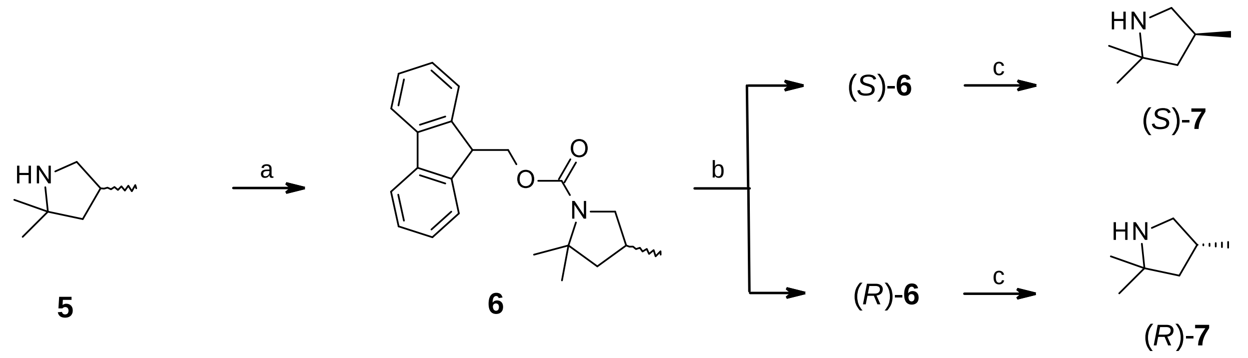

Scheme 2. Reagents and conditions: (a) Fmoc chloride, Na_2CO_3, H_2O, dioxane, rt; (b) chiral semi-preparative HPLC; (c) 2M NaOH, dioxane, rt.

9H-Fluoren-9-ylmethyl 2,2,4-trimethylpyrrolidine-1-carboxylate (6). A solution of Na_2CO_3 (0.276 g, 2.61 mmol) in H_2O (12 mL) was added to a mixture of 2,2,4-trimethylpyrrolidine hydrochloride (**5**, 0.355 g, 2.37 mmol) in dioxane (12 mL). The resulting mixture was cooled at 0 °C, 9-fluorenylmethoxycarbonyl (Fmoc) chloride (0.675 g, 2.61 mmol) was added and stirring was continued at rt for 24 h. The residue was taken in Et_2O (50 mL) and 0.5 M HCl (50 mL). The phase were partitioned and the water phase extracted with Et_2O (2 × 50 mL). The combined organic phase was washed with 0,5 M HCl (2 × 25 mL), brine (25 mL), dried over Na_2SO_4 and evaporated. Purification by flash chromatography (cyclohexane/EtOAc, 9:1 v/v) afforded **6** (0.62 g, 77%) as colorless sticky oil, as a 2:1 mixture of two rotamers. ^{1}H NMR (400 MHz, $CDCl_3$) δ 7.81 – 7.71 (m, 2H), 7.65 – 7.55 (m, 2H), 7.44 – 7.27 (m, 4H), 4.66 (d, J = 4.5 Hz, 1H, major rotamer), 4.33 (d, J = 4.6 Hz, 1H, minor rotamer), 4.39 – 4.28 (m, 1H), 4.28 – 4.19 (m, 1H), 3.77 – 3.68 (m, 1H, major rotamer), 3.68 – 3.59 (m, 1H, minor rotamer), 2.94 (t, J = 10.5 Hz, 1H, major rotamer), 2.82 (t, J = 10.7 Hz, 1H, minor rotamer), 2.28 (ddq, J = 17.9, 12.2, 6.5 Hz, 1H, major rotamer), 2.11 (ddt, J = 17.9, 12.1, 6.5 Hz, 1H, minor rotamer), 1.91 (dd, J = 12.3, 6.2 Hz, 1H, major rotamer), 1.73 (dd, J = 12.4, 6.1 Hz, 1H minor rotamer), 1.49 (s, 3H, major rotamer), 1.47 – 1.42 (m, 1H, major rotamer), 1.35 (s, 3H, major rotamer), 1.33 – 1.28 (m, 1H, minor rotamer), 1.06 (d, J = 6.5 Hz, 3H, major rotamer), 0.94 (d, J = 6.4 Hz, 1H, minor rotamer), 0.84 (s, 3H, minor rotamer), 0.79 (s, 3H, minor rotamer). UPLC-MS: t_R = 2.19 min (Apolar method). MS (ESI) m/z calcd for $C_{22}H_{26}NO_2$ $[M+1]^+$: 336.2, found: 336.3.

9H-Fluoren-9-ylmethyl (*4R*)-2,2,4-trimethylpyrrolidine-1-carboxylate ((*R*)-6) and **9H-fluoren-9-ylmethyl (*4S*)-2,2,4-trimethylpyrrolidine-1-carboxylate ((*S*)-6).** Compound **6** (0.32 g, 0.96 mmol) was purified under the chiral semi-preparative HPLC conditions described above *(please refer to synthetic materials and methods section)*. The fractions containing the pure enantiomers were evaporated in vacuo affording (*R*)-**6** (first eluted) and (*S*)-**6** (second eluted) as white solids. (*R*)-**6** (0.133 g, 41%): > 99.5% ee. t_R 27.62 min. (*S*)-**6** (0.115 g, 36%): > 99.5% ee. t_R 39.05 min. ^{1}H NMR, and UPLC-MS analyses were identical to those of the racemic mixture.

(*4R*)-2,2,4-Trimethylpyrrolidine hydrochloride ((*R*)-7). Compound (*R*)-**6** (0.13 g, 0.387 mmol) was dissolved in dioxane (4.2 mL) and 2 M NaOH (0.58 mL, 1.16 mmol) was added. The resulting mixture was stirred at rt for 21 h. 1 M HCl (25 mL) was added to the reaction mixture and the water phase was washed with Et_2O (3 × 25 mL). The organic phases were discarded and the water phase was evaporated affording (*R*)-**7** (0.12 g, quant.) as white solid. The product containing inorganic salts was used in the next step without any further purification. ^{1}H NMR (600 MHz, D_2O) δ 3.50 (dd, *J* = 11.8, 8.0 Hz, 1H), 2.91 (dd, *J* = 11.8, 9.5 Hz, 1H), 2.68 – 2.52 (m, 1H), 2.14 (dd, *J* = 13.2, 7.5 Hz, 1H), 1.54 (dd, *J* = 13.2, 10.4 Hz, 1H), 1.49 (s, 3H), 1.42 (s, 3H), 1.11 (d, *J* = 6.7 Hz, 3H).

(*4S*)-2,2,4-Trimethylpyrrolidine hydrochloride ((*S*)-7). Compound (*S*)-**6** (0.11 g, 0.331 mmol) was dissolved in dioxane (3.6 mL) and 2 M NaOH (0.50 mL, 0.993 mmol) was added. The resulting mixture was stirred at rt for 17 h. 1 M HCl (25 mL) was added to the reaction mixture and the water phase was washed with Et_2O (3 × 25 mL). The organic phases were discarded and the water phase was evaporated affording (*S*)-**7** (0.11 g, quant.) as white solid. The product containing inorganic salts was used in the next step without any further purification. ^{1}H NMR (600 MHz, D_2O) δ 3.50 (dd, *J* = 11.8, 8.0 Hz, 1H), 2.91 (dd, *J* = 11.8, 9.5 Hz, 1H), 2.68 – 2.52 (m, 1H), 2.14 (dd, *J* = 13.2, 7.5 Hz, 1H), 1.54 (dd, *J* = 13.2, 10.4 Hz, 1H), 1.49 (s, 3H), 1.42 (s, 3H), 1.11 (d, *J* = 6.7 Hz, 3H).

Synthesis of **VX-445**.

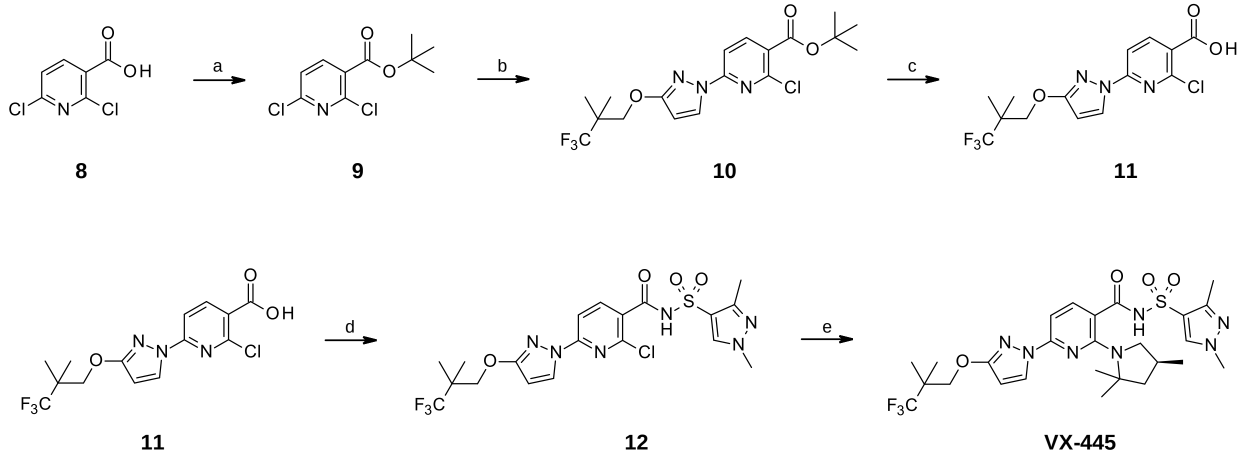

Scheme 3. Reagents and conditions: (a) Boc anhydride, DMAP, THF, rt; (b) **4**, K_2CO_3, DABCO, DMF, rt; (c) 6M HCl, dioxane, 85 °C; (d) 1,3-Dimethylpyrazole-4-sulfonamide, CDI, DBU, THF, rt; (e) (*S*)-**7**, K_2CO_3, DMSO, 130 °C.

***tert*-Butyl 2,6-dichloropyridine-3-carboxylate (9).** To a solution of 2,6-dichloropyridine-3-carboxylic acid (**8**, 1.0 g, 5.21 mmol) in tetrahydrofuran (20 mL) were added di-*tert*-butyl dicarbonate (1.70 g, 7.81 mmol) and 4-(dimethylamino)pyridine (0.32 g, 2.60 mmol). The mixture was stirred at rt for 24 h. 1 M HCl (40 mL) was added and the mixture stirred vigorously for 10 min. The product was extracted EtOAc (3 × 20 mL), washed with H_2O (20 mL), brine (20 mL), dried over Na_2SO_4 and concentrated. Purification by flash chromatography (cyclohexane/EtOAc, 98:2 v/v) afforded **9** (1.12 g, 86%) as colorless oil. ^{1}H NMR (400 MHz, $CDCl_3$) δ 8.04 (d, J = 8.0 Hz, 1H), 7.32 (d, J = 8.1 Hz, 1H), 1.60 (s, 9H). UPLC-MS: t_R = 2.49 min (Generic method). MS (ESI) m/z calcd for $C_{10}H_{12}Cl_2NO_2$ $[M+1]^+$: 248.0, found: 247.9.

***tert*-Butyl 2-chloro-6-[3-(3,3,3-trifluoro-2,2-dimethyl-propoxy)pyrazol-1-yl]pyridine-3-carboxylate (10).** To a solution of **4** (0.20 g, 0.97 mmol) and **9** (0.24 g, 0.97 mmol) in *N*,*N*-dimethylformamide (4.0 mL) were added K_2CO_3 (0.17 g, 1.26 mmol) and 1,4-diazabicyclo[2.2.2]octane (0.016 g, 0.145 mmol). The resulting mixture was stirred at rt for 72 h. The solution was poured into H_2O (50 mL) and extracted in EtOAc (5 × 50 mL). The combined organic phase was washed with H_2O (2 × 15 mL), brine (20 mL), dried over Na_2SO_4, and evaporated. Heptane (3 mL) was added to the crude product and evaporated four times. Purification by flash chromatography (cyclohexane/EtOAc, 98:2 v/v) afforded **10** (0.34 g, 84%) as white solid.

^{1}H NMR (400 MHz, DMSO-d_6) δ 8.43 (d, J = 2.8 Hz, 1H), 8.31 (d, J = 8.4 Hz, 1H), 7.76 (d, J = 8.4 Hz, 1H), 6.25 (d, J = 2.8 Hz, 1H), 4.27 (s, 2H), 1.56 (s, 9H), 1.24 (s, 6H). UPLC-MS: t_R = 2.55 min (Apolar method). MS (ESI) m/z calcd for $C_{18}H_{22}ClF_3N_3O_3$ $[M+1]^+$: 420.1, found: 420.0.

2-Chloro-6-[3-(3,3,3-trifluoro-2,2-dimethyl-propoxy)pyrazol-1-yl]pyridine-3-carboxylic acid (11). 6 M HCl (2.7 mL) was added to a solution of **10** (0.68 g, 1.63 mmol) in 1,4-dioxane (15 mL) and stirred at 85 °C for 13 h. The solution was poured into H_2O (75 mL) and a precipitate occurred. The product was extracted with EtOAc (3 × 50 mL). The combined organic phase was washed with brine (25 mL), dried over Na_2SO_4, and evaporated to give **11** (0.58 g, 97%) as white solid. The product was used in the next step without any further purification. ^{1}H NMR (400 MHz, DMSO-d_6) δ 8.44 (d, J = 2.8 Hz, 1H), 8.39 (dd, J = 8.4, 1.1 Hz, 1H), 7.76 (d, J = 8.4 Hz, 1H), 6.25 (d, J = 2.8 Hz, 1H), 4.27 (s, 2H), 1.24 (s, 6H). UPLC-MS: t_R = 1,73 min (Generic method). MS (ESI) m/z calcd for $C_{14}H_{14}ClF_3N_3O_3$ $[M+1]^+$: 364.1, found: 363.9.

2-Chloro-*N*-(1,3-dimethylpyrazol-4-yl)sulfonyl-6-[3-(3,3,3-trifluoro-2,2-dimethyl-propoxy)pyrazol-1-yl]pyridine-3-carboxamide (12). A flame-dried flask was charged with a solution of **11** (0.57 g, 1.58 mmol) and 1,1′-carbonyldiimidazole (0.31 g, 1.90 mmol) in anhydrous tetrahydrofuran (7.0 mL) under nitrogen and stirred for 1 h. 1,3-dimethylpyrazole-4-sulfonamide (0.33 g, 1.90 mmol) and 1,8-diazabicyclo[5.4.0]undec-7-ene (0.28 mL, 1.90 mmol) were added and the reaction mixture was stirred overnight at rt. The volatiles were evaporated. The residue was taken in EtOAc (50 mL), washed with 0,5 M HCl (50 mL) and the water phase was further extracted with EtOAc (2 × 50 mL). The combined organic phase was washed with brine (30 mL), dried over Na_2SO_4 and evaporated. Purification by flash chromatography (CH_2Cl_2 and then CH_2Cl_2 /MeOH 97:3 v/v) afforded **12** (0.73 g, 88%) as white solid. ^{1}H NMR (400 MHz, DMSO-d_6) δ 12.71 (s, 1H), 8.42 (d, J = 2.9 Hz, 1H), 8.37 (s, 1H), 8.09 (d, J = 8.4 Hz, 1H), 7.73 (d, J = 8.3 Hz, 1H), 6.24 (d, J = 2.9 Hz, 1H), 4.26 (s, 2H), 3.83 (s, 3H), 2.34 (s, 3H), 1.23 (s, 6H). UPLC-MS: t_R = 1,81 min (Generic method). MS (ESI) m/z calcd for $C_{19}H_{21}ClF_3N_6O_4S$ $[M+1]^+$: 521.1, found: 520.9.

***N*-(1,3-Dimethylpyrazol-4-yl)sulfonyl-6-[3-(3,3,3-trifluoro-2,2-dimethyl-propoxy)pyrazol-1-yl]-2-[(*4S*)-2,2,4-trimethylpyrrolidin-1-yl]pyridine-3-carboxamide (VX-445).** Compound **12** (0.057 g, 0.11 mmol), (*S*)-**7** (0.33 mmol), and K_2CO_3 (0.091 g, 0.66 mmol) were combined in anhydrous DMSO (0.4 mL) under nitrogen and stirred at 130 °C for 20 h. The reaction mixture

was poured into H_2O (30 mL), the pH was adjusted to 3-4 by adding 2 M HCl and extraction with Et_2O (3 × 25 mL) was performed. The combined organic phase was washed with brine (2 × 20 mL), dried over Na_2SO_4 and evaporated. Purification by flash chromatography (cyclohexane/EtOAc, 7:3 v/v) afforded an impure product, which was further purified by flash chromatography (CH_2Cl_2/MeOH 99:1 v/v) to give pure **VX-445** (0.030 g, 46%) as white solid. ^1H NMR (400 MHz, DMSO-d_6) δ 12.33 (s, 1H), 8.34 (s, 1H), 8.21 (d, J = 2.7 Hz, 1H), 7.73 (d, J = 8.2 Hz, 1H), 6.93 (d, J = 8.2 Hz, 1H), 6.16 (d, J = 2.7 Hz, 1H), 4.23 (s, 2H), 3.80 (s, 3H), 2.57 (t, J = 10.4 Hz, 1H), 2.48 – 2.39 (m, 1H), 2.32 (s, 3H), 2.25 – 2.08 (m, 1H), 1.87 (dd, J = 12.0, 5.6 Hz, 1H), 1.56 (s, 3H), 1.53 (s, 3H), 1.42 (t, J = 12.1 Hz, 1H), 1.23 (s, 6H), 0.81 (d, J = 6.3 Hz, 3H). ^{13}C NMR (101 MHz, DMSO-d_6) δ 165.2, 164.0, 152.3, 148.9, 146.7, 141.5, 136.4, 128.6 (q, J = 285.4 Hz), 128.4, 117.3, 111.2, 96.2, 95.3, 70.9, 64.0, 57.7, 50.8, 40.8 (q, J = 24.3 Hz), 38.7, 29.7, 26.4, 25.0, 18.1, 16.4, 12.0. UPLC-MS: t_R = 2,51 min (Generic method). MS (ESI) m/z calcd for $C_{26}H_{35}F_3N_7O_4S$ $[M+1]^+$: 598.2, found: 598.4. $[\alpha]_D^{27}$ – 45.682 (c 0.199, $CHCl_3$). Chiral HPLC: > 99,4% ee, t_R = 24.25 min.
